# Acquisitions of behavioral health treatment facilities from 2010 to 2021

**DOI:** 10.1093/haschl/qxae080

**Published:** 2024-06-04

**Authors:** Ben Thornburg, Emma B McGinty, Julia Eddelbuettel, Alene Kennedy-Hendricks, Robert T Braun, Matthew D Eisenberg

**Affiliations:** Center for Mental Health and Addiction Policy, Department of Health Policy and Management, Johns Hopkins University, Baltimore, MD 21205, United States; Livingston Farrand Professor of Public Health, Division of Health Policy and Economics, Department of Population Health Sciences, Weill Cornell Medicine, New York, NY 10065, United States; Harvard University, PhD Program in Health Policy, Cambridge, MA 02138, United States; Center for Mental Health and Addiction Policy, Department of Health Policy and Management, Johns Hopkins University, Baltimore, MD 21205, United States; Department of Population Health Sciences, Weill Cornell Medicine, New York, NY 10065, United States; Center for Mental Health and Addiction Policy, Department of Health Policy and Management, Johns Hopkins University, Baltimore, MD 21205, United States

**Keywords:** mental health, substance use, market consolidation, private equity

## Abstract

Private equity (PE) and other for-profit ownership of behavioral health (mental health and substance use) treatment facilities have become increasingly prevalent, but data on these acquisitions are not readily available. In this study, we describe a novel database that contains information on the universe of behavioral health acquisitions that occurred between 2010 and 2021. We found that the frequency of behavioral health facilities involved in acquisitions increased substantially, from 32 facilities in 2010 to 1330 in 2021. The total number of facilities involved in acquisitions was 2806. Most of these facilities provided outpatient services only (*N* = 2073) and offered only mental health services (*N* = 1428). Private equity-backed acquisitions accounted for around 60% of all acquisition activity (*N* = 1678 facilities PE, *N* = 1128 facilities other for-profit). 25% of acquired facilities were located within 20 miles of one another (*N* = 561), 50% occurred within 80 miles (*N* = 1403), and 75% occurred within 319 miles (*N* = 2104). Future research should evaluate the effects of this consolidation on behavioral healthcare access, quality, spending, and patient outcomes.

## Introduction

Private equity (PE) and other forms of for-profit ownership, both private and public, are increasing across the US healthcare system, with unclear implications for patient health.^1^ The fragmented nature of many behavioral health (mental health and substance use disorder) facilities make them prime targets for acquisition; in other healthcare sectors, investors often add-value by creating efficiencies via consolidation and by leveraging economies of scale.^2,3^ Relative to other for-profit investors, PE firms are of particular interest for several reasons; they tend to promise significantly above average financial returns, they generally finance acquisitions by leveraging considerable debt, and they typically aim to sell acquired facilities on a brief, 3-7 year timeline.^4,5^

Mental illness and substance use disorder are leading causes of morbidity and mortality in the United States, and most people with these conditions do not receive high-quality treatment.^6^ Understanding acquisitions in this sector is a crucial first step to assessing the implications of these acquisitions for behavioral health treatment. As such, the objective of this study is to describe acquisitions in the behavioral health sector that occurred between 2010 and 2021. Additionally, investors may take levels of care (outpatient, inpatient, intensive outpatient, residential, or a combination) into account when making acquisition decisions, so we explore these heterogeneities within the sector. For example, economies of scale may arise from the acquisition of facilities that provide the same levels of care, or investors may be incentivized to construct a fuller continuum of care by acquiring facilities that provide different levels of care.

**Figure 1.**
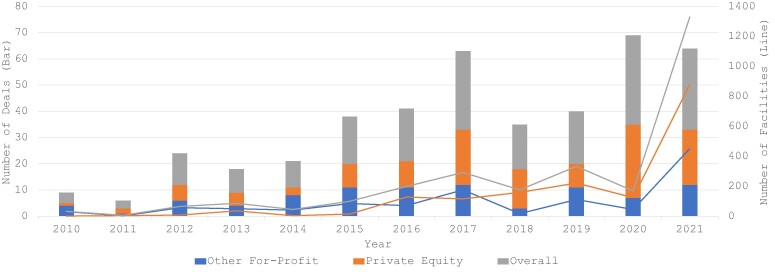
Number of behavioral health acquisitions and number of facilities involved by deal class between 2010 and 2021. Source: Authors’ Analysis of a novel database of behavioral health acquisitions, comprised of PitchBook capital market data (2010-2021) supplemented with information from structured web searches. Notes: Deals (left y-axis, line chart) are defined as an acquisition by a private equity or other for-profit investor, which involve one or more facilities (right y-axis, bar chart). Deal class is defined by whether the acquisition was by a private equity firm or other for-profit firm. “Overall” is the sum of private equity and other for-profit acquisitions, or the treatment facilities they involve, respectively. All statistics reported are simple aggregates by year.

Using a novel database on acquisitions in behavioral healthcare, we identified 4 acquisitions involving 32 behavioral health treatment facilities in 2010, with the number of acquisitions growing to 31 involving 1330 facilities in 2021, which is shown in Figure 1. This growth was observed among acquisitions backed by both PE firms and other for-profit firms. The 2021 purchases of a behavioral health system and a chain of recovery centers by a PE firm and another for-profit firm, respectively, drove a large increase in the number of facilities involved in acquisitions in that year.

## Data and methods

Industry reports and research on merger & acquisition activity suggest that PE and other for-profit acquisitions of behavioral health treatment facilities have become increasingly common over the past decade, though comprehensive information on this market activity is not readily available.^2,3,7,8^ To address this lack of data, we constructed a database of behavioral health acquisitions that occurred between 2010 and 2021.

We identified acquisitions using data from PitchBook, Inc, a company which tracks and records capital market activity across multiple industries from sources, such as SEC filings, reports obtained from general and limited partners, web scraping, and Freedom of Information Act requests. These data have been used extensively in research on healthcare acquisitions and are consistent with competing data suppliers.^9-14^ In particular, Brown et al.^15^ conducted an analysis of PitchBook and three other leading commercial data sources and found that despite differences in data collection methodologies, the four resulting datasets produced similar lists of acquisitions, suggesting that each likely provide an unbiased accounting of PE activity. To isolate acquisitions in the behavioral healthcare sector, a team of two researchers independently reviewed all unique descriptors of deals and discarded those that were unrelated to behavioral healthcare, defined as mental health or substance use care. Inter-coder agreement was 98%, and the residual was resolved through discussion with the project directors. The set of descriptors used to identify behavioral healthcare are available in the supplement (Table S1).

We then supplemented these data with primary data that was collected and organized using a three-step process. First, we used deal synopses detailed in the PitchBook data to determine the names of firms involved in acquisitions. Second, we determined the geographic locations of all relevant facilities by manually visiting each of their websites and collecting all facility location information provided. Third, we verified the type (mental health, substance use treatment, both) and levels (outpatient, inpatient, residential, intensive outpatient, multiple) of care available at each location. We used Internet Archive's Wayback Machine and date-restricted web searches to ensure these supplemental data were tied to the time of acquisition, as types and levels of care provided can change following acquisition.

We summarized acquisitions overall and stratified by deal class (PE/other for-profit). We measured the proximity of facilities to one another using the zip centroids associated with each facility's geographic location. We then calculated the number of facilities involved in acquisitions per 1000 individuals by hospital reference region, using denominators from the 2021 American Community Survey. Next, we used directories from the National Survey of Substance Abuse Treatment Services and the National Mental Health Services Survey to generate denominators for behavioral health treatment centers. Finally, we computed the proportion of these centers involved in acquisitions by care type, level, and overall. All analyses were conducted in R version 4.3.1.

Our study is limited by the fact that most (∼89%) of the acquisitions we described involve privately held firms which are subject to few disclosure requirements. Thus, it is impossible to know with certainty whether the Pitchbook Data captures every acquisition of a behavioral health facility. As noted previously, however, prior research substantiates the utility of the Pitchbook data.^8,9^ Additionally, though a well-established approach in the literature, we rely on archived websites, time-restricted searches, and press releases to construct our supplemental data.^9-13^

## Results

**Figure 2.**
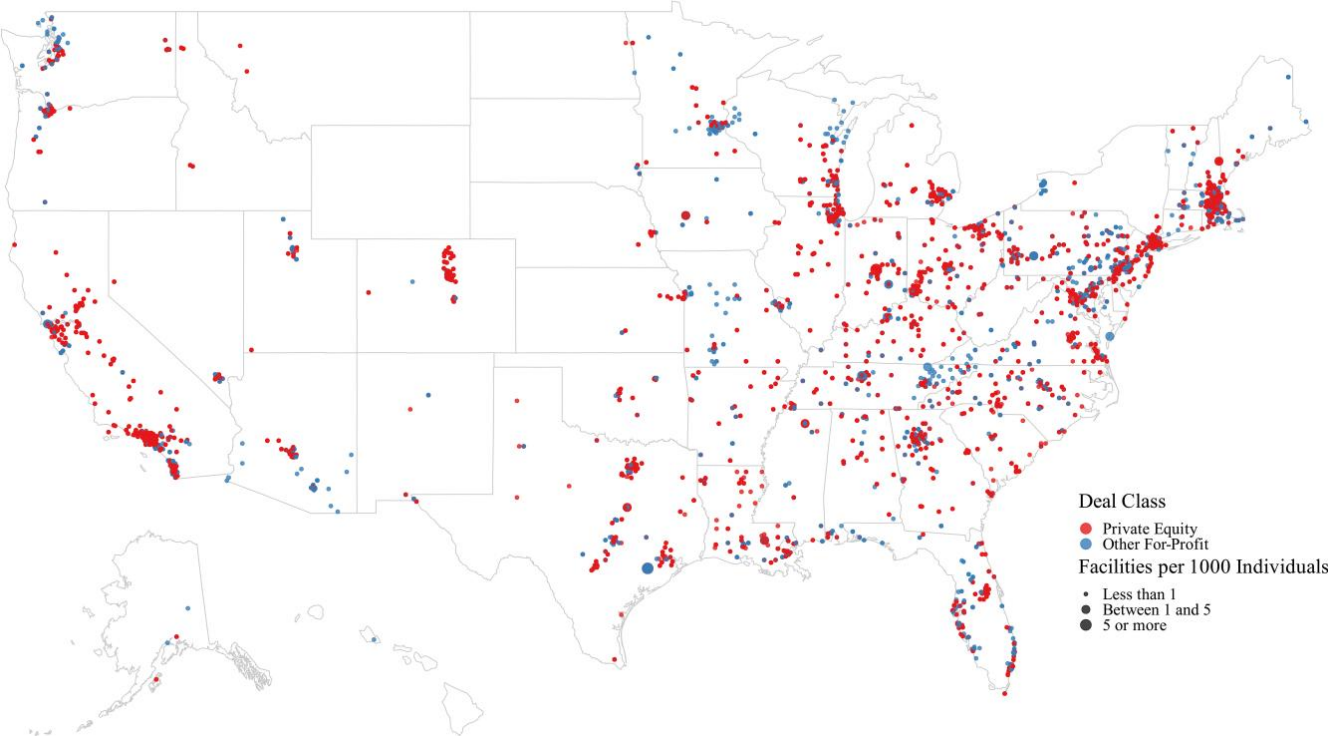
Geographic distribution of behavioral health facilities involved in acquisitions per 1000 individuals by hospital referral region 2010-2021. Source: Authors’ Analysis of a novel database of behavioral health acquisitions, comprised of PitchBook capital market data (2010-2021) supplemented with information from structured web searches. The 2021 American Community Survey Was Used To Compute Population Proportions. Notes: Each dot represents one or more facilities involved in an acquisition by a private equity or other for-profit firm, the size of the dot represents the number of facilities involved in acquisitions within a given hospital referral region, per 1000 people residing in that region according to the 2021 American Community Survey. The data are described cross-sectionally, in that each dot represents a facility or facilities acquired at any point between 2010 and 2021.

Between 2010 and 2021, there were 208 acquisitions involving 2806 unique behavioral health treatment facilities. Specifically, PE firms engaged in 119 deals involving 1657 facilities. Other for-profit firms engaged in 89 deals that involved 1149 facilities; the majority of these, 67 deals involving 696 facilities, were backed by privately held firms and the remainder, 23 deals involving 453 facilities, were backed by publicly traded firms. Figure 2 displays the geographic distribution of facilities involved in acquisitions during the study period as the number of acquisition-involved facilities per 1000 individuals by hospital referral region. 25% of facilities acquired in a given deal were located within 20 miles of one another (*N* = 561), 50% were within 80 miles (*N* = 1403), and 75% were within 319 miles (*N* = 2104).

**Figure 3.**
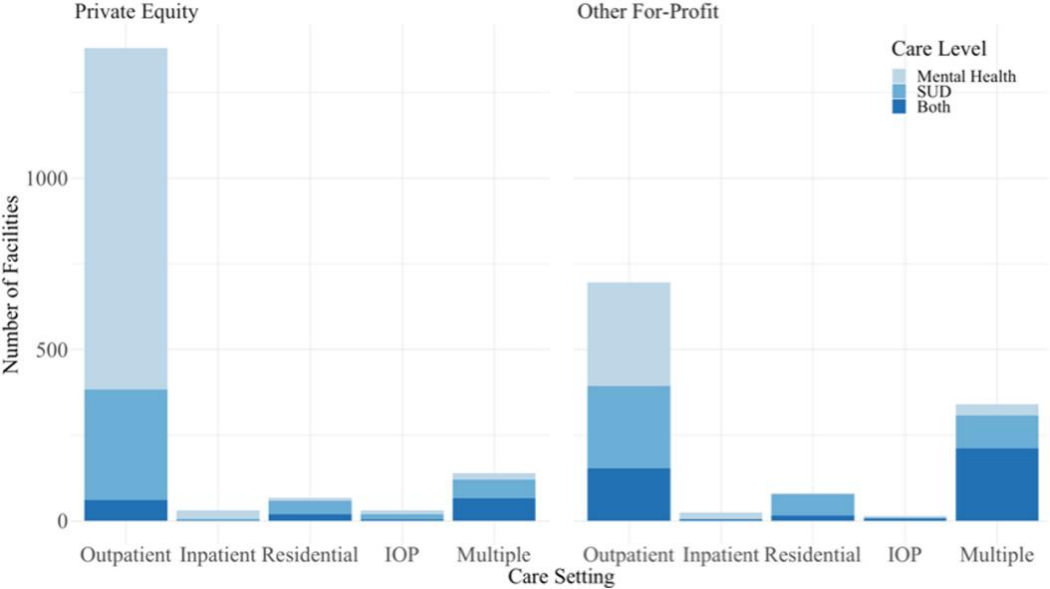
Levels and types of care provided by behavioral health facilities involved in an acquisition between 2010 and 2021. Source: Authors’ Analysis of a novel database of behavioral health acquisitions, comprised of PitchBook capital market data (2010-2021) supplemented with information from structured web searches. Notes: The three care levels are mutually exclusive; facilities providing both mental healthcare and SUD care are counted only once, in the “Both” group. Similarly, within each panel, the bars are mutually exclusive; facilities providing only one level of care are counted in the left four bars, where facilities providing at least two levels of care are counted only in the rightmost bar. The data are described cross-sectionally, in that bars describe the number of facilities acquired at any point between 2010 and 2021. All statistics reported are simple aggregates by care level. Deal class is defined by whether the facility was acquired by a private equity firm or other for-profit firm.

Figure 3 reports information on the types and levels of services provided at facilities involved in acquisitions between 2010 and 2021, showing that overall, most facilities provided outpatient mental health services (PE *N* = 997 facilities, other for-profit *N* = 302 facilities). Facilities providing intensive outpatient services (IOP) were involved in the fewest acquisitions (PE *N* = 31, other for-profit *N* = 14), followed by inpatient services (PE *N* = 32, other for-profit *N* = 23), residential care (PE *N* = 74, other for-profit *N* = 75), multiple care levels (PE *N* = 150, other for-profit *N* = 325), and facilities providing outpatient services were involved in the most acquisitions (PE *N* = 1,382, other for-profit *N* = 691). Facilities involved in PE backed acquisitions most often provided mental health services (*N* = 1065), while those involved in other for-profit acquisitions most often provided treatment for both mental health and substance use disorders (*N* = 385).

Overall, an estimated 7.0% (4.1% PE, 2.9% other for-profit) of all behavioral health treatment facilities were involved in an acquisition between 2010 and 2021. 13.1% of all facilities providing mental health services, 5.9% of all facilities providing SUD services, and 6.0% of all facilities providing both mental health and SUD services between 2010 and 2021 were involved in an acquisition. Moreover, 18.1% of all facilities providing outpatient services, 2.5% of those providing inpatient services, 3.6% of those providing residential services, and 2.3% of those providing IOP were involved in an acquisition between 2010 and 2021.

We also found that almost all (99%) facilities that provide mental health services were acquired by an investor that had previously acquired at least one facility that also provided mental health services (Figure S1). (To access the appendix, click on the Details tab of the Article online.) This pattern was also consistent for facilities that provided substance use treatment (98%), and across all care levels (Residential 953%, Outpatient 98.99%, Inpatient 78.18%, and IOP 82.22%). Additionally, we found that the number of investors entering the market over the study period was generally increasing (Figure S2). We also characterized the distribution of facilities acquired per acquisition. The median number of facilities acquired per acquisition was one, and the mean was 2 (Figure S3).

## Discussion

Acquisition of behavioral health treatment facilities by PE and other for-profit firms increased substantially from 2010 to 2021 in both the frequency of acquisitions and the number of facilities they involved. An estimated 7% of all behavioral health treatment facilities were involved in an acquisition between 2010 and 2021.^16^

This study adds to a burgeoning literature on mergers and acquisitions in the behavioral health sector. In particular, a recent study by Zhu et al. explored the penetration of PE ownership across outpatient and residential behavioral health treatment facilities by state between 2012 and 2023, and found an overall penetration rate of between 6% and 7%, where some states had a substantially higher rate than others.^10^ Our results were similar, indicating that these patterns are consistent across different study periods, additional levels of care, and other for-profit investors. Additionally, our findings complement previous qualitative research that highlighted features of the behavioral healthcare landscape attracting interest from investors, including (1) fragmentation; behavioral healthcare is much less connected than other healthcare sectors, making it possible to generate economies of scale via the consolidation of facilities, (2) recent policy shifts, including the Affordable Care Act of 2010 and the Mental Health Parity and Addiction Equity Act of 2008, expanding insurance coverage for behavioral health conditions, and (3) decreasing stigma around obtaining behavioral healthcare, which has contributed to growing demand for services. Furthermore, investors generally target one geographic area at a time, and, both PE and other for-profit investors prefer to acquire facilities that provide the same types (mental health, substance use, or both) and levels of care (outpatient, inpatient, residential, intensive outpatient, or multiple) as facilities they have previously acquired.

We found that PE firms that operate in the behavioral health sector adopt a “platform and roll-up” approach that is consistent with PE activity documented in other sectors.^1^ To use a representative example from our data, in 2017 one PE firm acquired an operator of several outpatient opioid use disorder treatment facilities located across the Southeast. Over the following years, the PE firm acquired several other operators of opioid use disorder treatment facilities that were also located in the Southeast. The initial acquisition (the “platform”) established a foundation for the PE firm's future investments in opioid use disorder care, which was then expanded via the addition of facilities from the subsequent acquisitions. In most cases, these “roll-up” facilities will take on the branding and mission of the platform.

These patterns are consistent with a “market power” strategy. For example, an investor that owns the majority of the outpatient mental health facilities in a geographic area will retain more influence in that market than if they were to have a diverse portfolio of facilities administering differing care types and levels across the country. While the latter is arguably more insulated from risk, it seems possible that in this context, investors would deprioritize risk-avoidance in their strategies given broad evidence of excess demand for behavioral health services across the country.^17^ There is still much to learn about how PE and other for-profit firms make decisions, particularly over time as the market for behavioral health treatment facilities becomes more crowded.

If investors can cultivate market power in this manner, then it is possible that prices and utilization of services would increase, a phenomenon that has been well documented in other healthcare sectors.^1^ That said, it remains an empirical question whether and how these acquisitions affect behavioral healthcare access, utilization, quality, and spending. Investigating these questions is critical given how quickly facilities are being consolidated and the high degree of unmet patient need for behavioral health care.^18^ In addition to considering the potentially unique effects of PE-backed acquisitions, it will be important to study other for-profit acquisitions by whether they are publicly traded vs privately held. A recent study suggests that relative to acquisitions by PE firms and other for-profit privately held firms, acquisitions by publicly traded, for-profit firms may have larger effects on care delivery, potentially because publicly traded for-profit firms need to hit quarterly earnings, which adds pressure to increase profits in ways that may not benefit patients.^19^

## Conclusion

We found that the frequency of acquisitions in the behavioral health sector increased substantially between 2010 and 2021. Future research should evaluate the effects of these acquisitions on behavioral healthcare access, quality, spending, and patient outcomes.

## Supplementary Material

qxae080_Supplementary_Data

## Data Availability

Data are available upon request.
