# Supplementary material for: Acquisitions of behavioral health treatment facilities from 2010 to 2021
Source: Health Aff Sch. 2024 Jun 4;2(7):qxae080. doi: 10.1093/haschl/qxae080 (PMC11235319; doi:10.1093/haschl/qxae080)
Supplement: qxae080_Supplementary_Data [file qxae080_supplementary_data.zip › Appendix materials HA 51624.docx]

Supplemental Figure 1: Proportion of Acquisitions in which the Acquired Facility was Consistent with the Levels or Types of Care of Facilities Owned by the Acquirer

Notes: The denominator used to compute these proportions was the total number of facilities acquired by the private equity firm or corporation over the study period (2010-2021) by whether the acquired facility provided residential, outpatient, inpatient, intensive outpatient care, and whether it offered care for substance use disorder or mental health conditions. The numerator was an aggregation of indicators equal to one if the marginal acquired facility provided residential, outpatient, inpatient, intensive outpatient care, and separately, whether it offered care for substance use disorder or mental health conditions. The resulting proportions then represent the relative diversity of acquisitions by private equity firms and corporations, by measuring how often they deviate from acquiring facilities that provide a specific type and level of care. The data are described cross-sectionally, in that bars describe the number of facilities acquired at any point between 2010 and 2021.


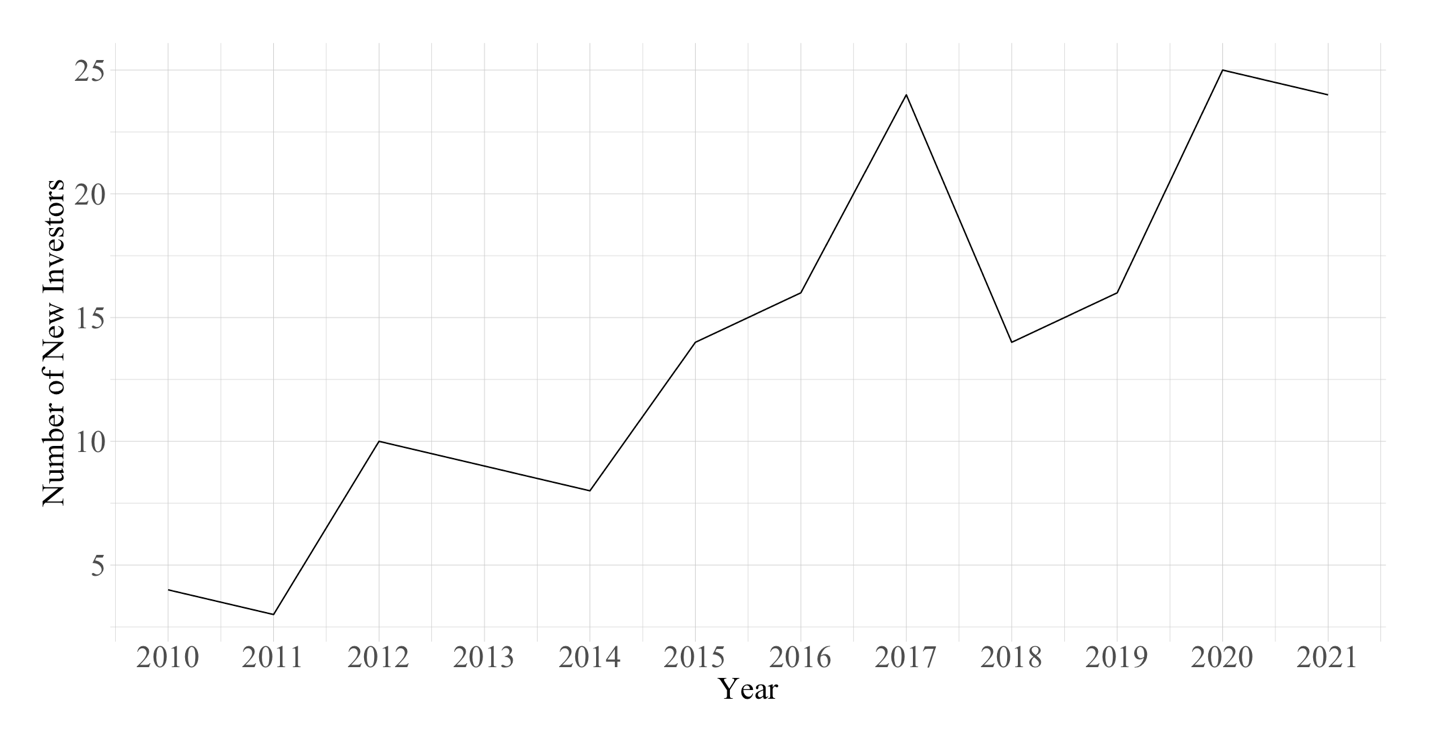
Supplemental Figure 2: Number of Investors Involved in Behavioral Health Acquisitions 2010-2021

**
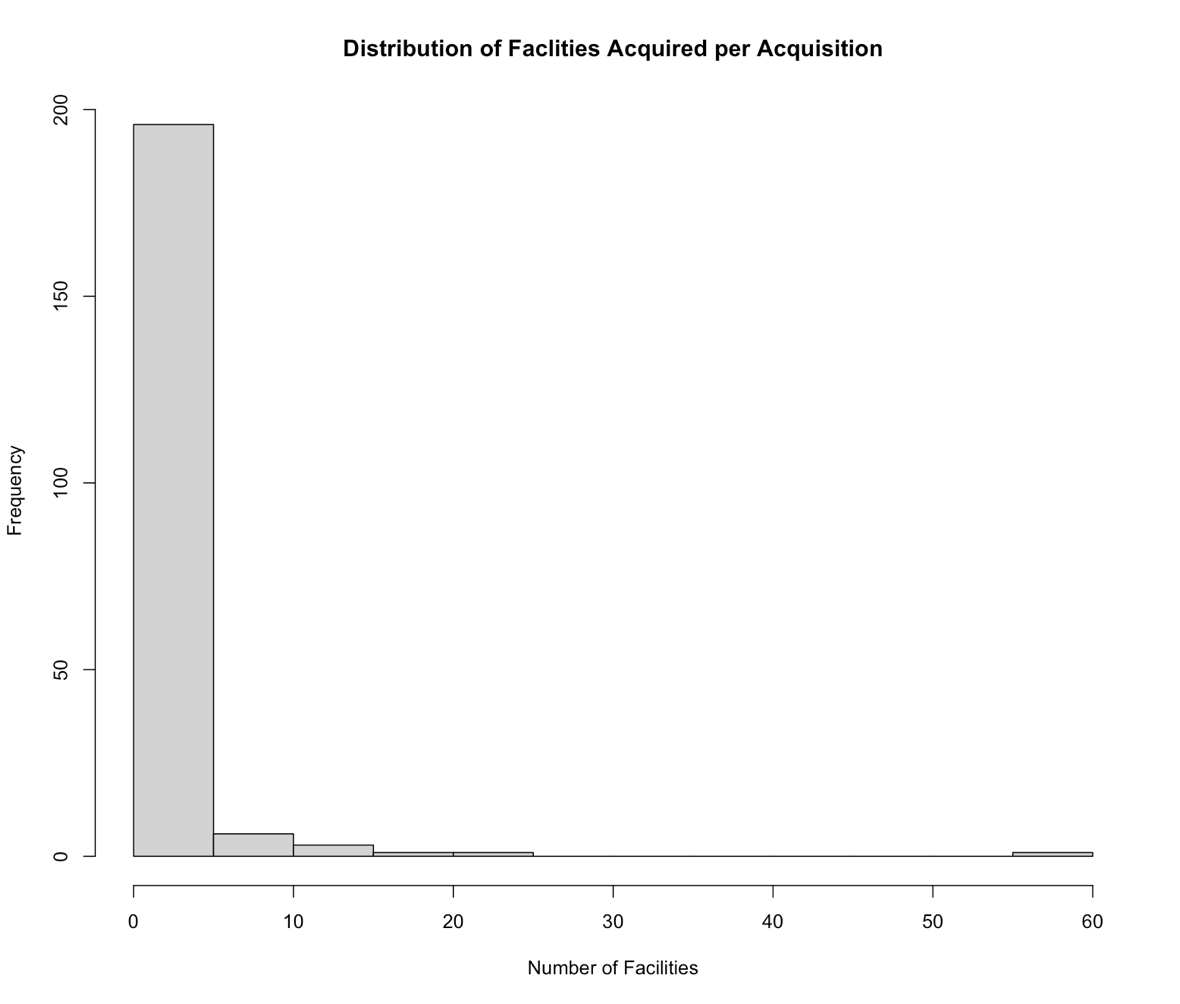
**Supplemental Figure 3: Distribution of Facilities Acquired per Acquisition

Supplemental table 1:

| **Keyword** |
| --- |
| aac |
| aba |
| aba center |
| aba center operator |
| aba platform company |
| aba program |
| aba service |
| aba services |
| aba therapy |
| aba therapy center |
| aba therapy services |
| aba tools |
| aba training system |
| aba treatment |
| aba treatment programs |
| abuse counseling |
| abuse management |
| abuse treatment |
| abuse treatment industry |
| abuse treatment programs |
| abuse treatment services |
| abuse-deterrence |
| academically focused substance use disorder treatment |
| accelerated detox |
| acetaminophen |
| acetaminophen tablets |
| ache relief |
| achieve lasting sobriety |
| acupuncture clinic |
| acupuncture clinic operator |
| acupuncture for chronic pain |
| acupuncture therapy |
| acupuncture treatment |
| acute mental healthcare |
| acute migraine drug |
| acute pain |
| acute pain management |
| acute pain recovery |
| acute pain service |
| acute pain therapy |
| acute pain treatment |
| acute psychiatric care |
| acute psychiatric treatment |
| acute respiratory distress syndrome |
| addicted people |
| addiction |
| addiction aftercare |
| addiction assistance |
| addiction care |
| addiction care services |
| addiction center |
| addiction clinic |
| addiction control |
| addiction counseling |
| addiction counseling service |
| addiction cure |
| addiction disease |
| addiction disorder |
| addiction disorder treatment |
| addiction education |
| addiction evaluation |
| addiction healing |
| addiction healing treatment |
| addiction issues |
| addiction management |
| addiction medication |
| addiction medicine |
| addiction medicine services |
| addiction peer application |
| addiction prevention |
| addiction prevention platform |
| addiction prevention therapy |
| addiction problem |
| addiction process |
| addiction recovery |
| addiction recovery application |
| addiction recovery assistance |
| addiction recovery center |
| addiction recovery platform |
| addiction recovery program |
| addiction recovery services |
| addiction recovery support |
| addiction recovery tools |
| addiction recovery treatment |
| addiction rehabilitation |
| addiction research |
| addiction research program |
| addiction service |
| addiction specialist |
| addiction therapeutics |
| addiction therapy |
| addiction treatment |
| addiction treatment center |
| addiction treatment channel |
| addiction treatment clinic |
| addiction treatment connection |
| addiction treatment consulting |
| addiction treatment drugs |
| addiction treatment facility |
| addiction treatment firm |
| addiction treatment industry |
| addiction treatment network |
| addiction treatment network channel |
| addiction treatment platform |
| addiction treatment programs |
| addiction treatment provider |
| addiction treatment research |
| addiction treatment service |
| addiction treatment support |
| addiction treatment testing |
| addiction wellness |
| addictions clinic |
| addictions counselors |
| addictive agent |
| addictive disease |
| addictive disorder |
| addition treatment |
| adenosine pharmaceutical |
| adenosine receptor |
| adenosine-based |
| adhd |
| adhd assessment |
| adhd awareness |
| adhd detection |
| adhd diagnostic test |
| adhd diagnostics |
| adhd drug developer |
| adhd drugs |
| adhd education platform |
| adhd patient treatment |
| adhd prevention |
| adhd program |
| adhd providers |
| adhd test |
| adhd therapy |
| adhd tools |
| adhd treatment |
| adhd treatment platform |
| adolescent addiction treatment |
| adolescent depression |
| adolescent iop/php service |
| adolescent mental health |
| adolescent mental health treatment |
| adolescent mental wellness |
| adolescent psychiatric hospital |
| adolescent psychiatry |
| adolescent psychotherapy |
| adolescent therapy |
| adrenergic blocker |
| adrenoceptor agonist |
| adult counseling |
| adult day care |
| adult iop/php service |
| adult mental illness |
| adult outpatient therapy |
| adult psychiatry |
| adult psychotherapy |
| adult therapy center |
| advances mental health |
| adventure based counseling |
| affordable therapy |
| ahss therapy process |
| ai-based brain training |
| alcohol |
| alcohol abuse |
| alcohol abuse center |
| alcohol abuse treatment |
| alcohol addiction |
| alcohol addiction recovery |
| alcohol addiction rehab |
| alcohol addiction treatment |
| alcohol and drug treatment facility |
| alcohol and drugs detoxification |
| alcohol breathalyzer |
| alcohol consumption reduction |
| alcohol consumption track |
| alcohol consumption tracking |
| alcohol counseling |
| alcohol dependence |
| alcohol dependency |
| alcohol detectors |
| alcohol detox |
| alcohol detoxification |
| alcohol drinking |
| alcohol intervention |
| alcohol level testing |
| alcohol recovery |
| alcohol reduction |
| alcohol rehab |
| alcohol rehab center |
| alcohol rehabilitation |
| alcohol rehabilitation center |
| alcohol rehabilitation service |
| alcohol screening |
| alcohol screening product |
| alcohol sensor |
| alcohol telehealth |
| alcohol test |
| alcohol testing |
| alcohol testing device |
| alcohol testing product |
| alcohol testing services |
| alcohol tracker |
| alcohol treatment |
| alcohol treatment center |
| alcohol treatment programs |
| alcohol treatment services |
| alcohol use disorder |
| alcohol use disorder platform |
| alcohol use disorder platform developer |
| alcohol use disorder platform provider |
| alcohol-testing program |
| alcoholic beverages |
| alcoholic hepatitis |
| alcoholic items |
| alcoholic treatment |
| alcoholism |
| alcoholism treatment |
| alkaloids manufacturer |
| alkaloids product |
| alpha-stim technology |
| alternate bilateral stimulation |
| alternative pain management |
| amnestic mild cognitive impairment |
| amputation pain healer |
| analgesia |
| analgesia tablets |
| analgesic |
| analgesic agent |
| analgesic clinical trials |
| analgesic components |
| analgesic drug |
| analgesic fundamentals |
| analgesic medicine |
| analgesic producer |
| analgesic product |
| analgesic property |
| analgesic research |
| analgesic therapy |
| anesthesia and pain management |
| anesthesia for pain management |
| anesthetic cream |
| anesthetic pain |
| anesthetic spray |
| ankle pain |
| ankle pain therapy |
| ankle pain treatment |
| anorexia |
| anorexia bulimia |
| anorexia drugs |
| anorexia nervosa |
| anorexia nervosa treatment |
| anorexia treatment |
| anti addiction drugs |
| anti depression drugs |
| anti depression therapy |
| anti depression treatment |
| anti doping |
| anti drugs |
| anti drugs clinic |
| anti pain drugs |
| anti-addiction |
| anti-addiction counseling |
| anti-addiction drugs |
| anti-addiction healthcare |
| anti-addiction therapy |
| anti-depressant |
| anti-depressant efficacy |
| anti-depressant market |
| anti-depression application |
| anti-depression drugs |
| anti-inflammation medicine |
| anti-inflammatory |
| anti-inflammatory compound |
| anti-inflammatory drug |
| anti-inflammatory effect |
| anti-inflammatory peptides |
| anti-inflammatory pharmaceutical |
| anti-inflammatory therapeutic |
| anti-inflammatory therapeutics |
| anti-inflammatory treatment |
| anti-pain therapy |
| anti-psychotic drugs |
| anti-smoking accessory |
| antidepressants |
| antidepressants drugs |
| antidrug medicne |
| antipsychotic drugs |
| anxiety |
| anxiety and depression |
| anxiety care |
| anxiety control |
| anxiety control product |
| anxiety counselling |
| anxiety cure |
| anxiety disorder |
| anxiety disorder therapy |
| anxiety disorders treatment |
| anxiety drugs |
| anxiety free device |
| anxiety management |
| anxiety medication |
| anxiety monitoring |
| anxiety overcome |
| anxiety relief |
| anxiety support |
| anxiety therapy |
| anxiety tracker |
| anxiety tracking |
| anxiety tracking device |
| anxiety treatment |
| anxiety treatment courses |
| anxiety treatment platform |
| apap tablets |
| applied behavior |
| applied behavior analysis |
| applied behavior analysis firm |
| applied behavior analysis program |
| applied behavior analysis service |
| applied behavior analysis service firm |
| applied behavior analysis therapy |
| applied behavior analysis therapy services |
| applied behavior analysis treatment |
| applied behavioral analysis |
| applied behavioral science |
| applied behaviour analysis therapy |
| applied behavioural analysis |
| aqua therapy |
| aquatic therapy |
| aquatic therapy clinic |
| aquatic therapy provider |
| aquatic therapy services |
| ards treatment |
| arketamine |
| art therapy |
| asd challenge |
| asd therapy |
| asd treatment center |
| asperger's syndrome |
| aspirin |
| aspirin tablets |
| attachment anxiety treatment |
| attachment disorder |
| attention deficit |
| attention deficit disorder |
| attention deficit hyperactivity disorder |
| audio therapy |
| auditory stimulation |
| autism |
| autism & aba services |
| autism and mental healthcare |
| autism and special needs |
| autism application |
| autism assessment platform |
| autism awareness |
| autism care |
| autism care center |
| autism care clinic |
| autism care program |
| autism care school |
| autism care services |
| autism care unit |
| autism center |
| autism center management software |
| autism child care |
| autism clinic |
| autism clinical services |
| autism community |
| autism counseling service provider |
| autism data library |
| autism day care |
| autism detection |
| autism device |
| autism diagnostic |
| autism diagnostics |
| autism discovery |
| autism disorder |
| autism disorder treatment |
| autism education |
| autism educational programs |
| autism family services |
| autism gene |
| autism health |
| autism kids |
| autism learning system |
| autism management |
| autism management platform |
| autism personalized treatment |
| autism playgroups |
| autism rehabilitation |
| autism remedies |
| autism research |
| autism safety assistive technology |
| autism school |
| autism service provider |
| autism services |
| autism skills center |
| autism software |
| autism spectrum |
| autism spectrum disorder |
| autism spectrum disorder care |
| autism spectrum disorder identification |
| autism spectrum disorder program |
| autism spectrum disorder treatment |
| autism support |
| autism support service |
| autism teacher |
| autism teaching |
| autism technology |
| autism test |
| autism therapeutics |
| autism therapy |
| autism therapy center |
| autism therapy provider |
| autism therapy system |
| autism training |
| autism treatment |
| autism treatment center |
| autism treatment planning |
| autism treatment platform |
| autism treatment sessions |
| autism wearable |
| autistic |
| autistic care |
| autistic child care |
| autistic children |
| autistic disorder |
| autistic medical aid |
| autistic risk score |
| autistic spectrum |
| autistic support |
| avoidant restrictive food intake disorder |
| ayurveda therapy |
| back pain |
| back pain condition |
| back pain control |
| back pain cure |
| back pain device |
| back pain drugs |
| back pain injection |
| back pain management |
| back pain program |
| back pain recovery |
| back pain relief product |
| back pain solution |
| back pain therapy |
| back pain treatment |
| back syndrome |
| backpain relief |
| baclofen injection |
| behavior altering chemical producer |
| behavior altering chemicals |
| behavior analysis |
| behavior analysis platform |
| behavior analysis program |
| behavior analysis service |
| behavior analysis therapy |
| behavior analysts |
| behavior analytics |
| behavior analytics platform |
| behavior assessment |
| behavior change |
| behavior change acceleration |
| behavior change analytics |
| behavior change coaching |
| behavior change counseling |
| behavior change platform |
| behavior change program |
| behavior consulting |
| behavior counseling |
| behavior data |
| behavior designing company |
| behavior disorder |
| behavior disorder center |
| behavior guidance |
| behavior health analysis |
| behavior health center |
| behavior health management |
| behavior imaging |
| behavior insights |
| behavior intervention |
| behavior intervention services |
| behavior journaling |
| behavior management |
| behavior management service |
| behavior management training |
| behavior modification |
| behavior modification product |
| behavior monitoring |
| behavior monitoring device |
| behavior patterns |
| behavior platform |
| behavior program |
| behavior reading platform |
| behavior science |
| behavior services |
| behavior support methodology |
| behavior testing |
| behavior therapy |
| behavior therapy services |
| behavior tracking |
| behavior tracking platform |
| behavior tracking tools |
| behavior-modification system |
| behavioral |
| behavioral abilities |
| behavioral analysis |
| behavioral analysts |
| behavioral analytics |
| behavioral analytics platform |
| behavioral analytics services |
| behavioral analytics tool |
| behavioral and mental health services |
| behavioral assessment |
| behavioral assessment service |
| behavioral care |
| behavioral care unit |
| behavioral challenge |
| behavioral change |
| behavioral change platform |
| behavioral change technology |
| behavioral clinical services |
| behavioral counseling |
| behavioral data |
| behavioral data analysis |
| behavioral data analytics |
| behavioral development |
| behavioral diagnostics |
| behavioral disability |
| behavioral disorder |
| behavioral disorder management |
| behavioral disorder therapeutic |
| behavioral disorder treatment |
| behavioral elements |
| behavioral examination |
| behavioral factors |
| behavioral health |
| behavioral health analysis |
| behavioral health analytics |
| behavioral health application |
| behavioral health artificial intelligence |
| behavioral health assessment |
| behavioral health care |
| behavioral health care center |
| behavioral health care service |
| behavioral health care unit |
| behavioral health center |
| behavioral health challenges |
| behavioral health clinic |
| behavioral health consultancy |
| behavioral health consultant |
| behavioral health consultation |
| behavioral health consulting |
| behavioral health data |
| behavioral health disorder |
| behavioral health ehr platform |
| behavioral health facilities |
| behavioral health hospital |
| behavioral health illness |
| behavioral health information |
| behavioral health institutions |
| behavioral health intervention |
| behavioral health issues |
| behavioral health management |
| behavioral health management service |
| behavioral health management software |
| behavioral health needs |
| behavioral health nonprofit group |
| behavioral health organization |
| behavioral health outcomes |
| behavioral health planning |
| behavioral health platform |
| behavioral health practice |
| behavioral health problems |
| behavioral health product |
| behavioral health program |
| behavioral health property |
| behavioral health providers |
| behavioral health rehabilitation services |
| behavioral health school |
| behavioral health sector |
| behavioral health services |
| behavioral health software |
| behavioral health solutions |
| behavioral health support |
| behavioral health system |
| behavioral health technology |
| behavioral health telemedicine |
| behavioral health therapy |
| behavioral health treatment |
| behavioral health-specific ehr platform |
| behavioral healthcare |
| behavioral healthcare campus |
| behavioral healthcare company |
| behavioral healthcare education |
| behavioral healthcare facility |
| behavioral healthcare management |
| behavioral healthcare organizations |
| behavioral healthcare platform |
| behavioral healthcare program |
| behavioral healthcare service |
| behavioral healthcare software |
| behavioral healthcare system |
| behavioral healthcare training |
| behavioral healthcare treatment |
| behavioral hospital |
| behavioral illness |
| behavioral informatics |
| behavioral insights |
| behavioral instruction |
| behavioral intelligence platform |
| behavioral intervention |
| behavioral issues |
| behavioral learning |
| behavioral management |
| behavioral medicine |
| behavioral mental health |
| behavioral model |
| behavioral neuroscience |
| behavioral outcome |
| behavioral pharmacology |
| behavioral problem |
| behavioral psychology |
| behavioral research |
| behavioral risk trend analysis |
| behavioral science |
| behavioral sciences technology |
| behavioral services |
| behavioral study |
| behavioral support |
| behavioral support service |
| behavioral support services |
| behavioral system |
| behavioral testing |
| behavioral therapy |
| behavioral therapy services |
| behavioral therapy tools |
| behavioral therapy training |
| behavioral therapy training service |
| behavioral training |
| behavioral traits |
| behavioral treatment |
| behavioral treatment support |
| behavioral weight loss |
| behavioralhealth |
| behaviour analysis |
| behaviour analytics |
| behaviour change support |
| behaviour monitoring |
| behaviour platform |
| behaviour program |
| behaviour tracker |
| behavioural change |
| behavioural change detection |
| behavioural health |
| behavioural health care |
| behavioural health issues |
| behavioural health platform |
| behavioural health services |
| behavioural health software |
| behavioural health support |
| behavioural health tech |
| behavioural healthcare |
| behavioural healthcare technology |
| behavioural problem |
| behavioural study |
| behavioural therapy |
| behavioural training |
| behavioural treatment |
| behavourial health |
| belly pain medicine |
| beta blocker |
| beta blocker treatment |
| beta-blockers |
| beta-blockers prescription |
| beta-caryophyllene |
| binge eating |
| binge eating disorder |
| bio-psychosocial assessment |
| bioelectric signaling |
| bioelectric technology |
| bioelectric therapy |
| bioelectronic therapy |
| bioelectronic treatment |
| bioelectronics devices |
| bipolar disorder |
| bipolar disorder care |
| bipolar disorder cure |
| bipolar disorder detection |
| bipolar disorder drugs |
| bipolar disorder scanning |
| bipolar disorder treatment |
| bipolar drugs |
| bipolar electrocautery |
| bipolar electrocautery device |
| bipolar episode |
| bipolar patients |
| bipolar treatment |
| blocks pain |
| blood alcohol |
| blood alcohol content |
| blood alcohol measuring |
| body aches |
| body massage services |
| body pain treatment |
| borderline personality disorder |
| botanical therapeutic |
| botanical therapy |
| botox therapy |
| botox treatment |
| brain development therapy |
| brain stimulation |
| brain stimulation development |
| brain stimulation device |
| brain stimulation lab |
| brain stimulation technology |
| brain stimulation therapy |
| brain stimulations |
| brain stimulator |
| brain therapy |
| brain training program |
| brain training software |
| bulimia |
| bulimia nervosa |
| bulimia treatment |
| buprenorphine |
| buprenorphine release |
| buprenorphine treatment |
| bursitis |
| bursitis therapy |
| cannabidiol medicine |
| cannabidiol sleep product |
| cannabidiol therapeutic |
| cannabinoid medicine |
| cannabinoid pharmaceuticals |
| cannabinoid therapeutics |
| cannabinoid therapy |
| cannabinoid treatment |
| cannabinoid treatment product |
| cannabinoid-based health product |
| cannabinoid-based therapeutics |
| cannabinoid-based therapy |
| cannabinoid-derived medicine |
| cannabinoid-modulating medicine |
| cannabinoids-based medicine |
| cannabis antidote |
| cannabis derived medicine |
| cannabis health care |
| cannabis healthcare |
| cannabis medical |
| cannabis medical benefit |
| cannabis medical consultation |
| cannabis medical dispensary |
| cannabis medical dosage |
| cannabis medical drugs |
| cannabis medical knowledge |
| cannabis medical product |
| cannabis medical products |
| cannabis medical research |
| cannabis medication |
| cannabis medication developer |
| cannabis medication development |
| cannabis medicine |
| cannabis medicine manufacturer |
| cannabis medicine retailer |
| cannabis pain relief |
| cannabis pain treatment |
| cannabis pharmaceutical |
| cannabis pharmaceutical product |
| cannabis pharmaceuticals |
| cannabis plant health |
| cannabis plant medicine |
| cannabis therapeutic |
| cannabis therapeutic product |
| cannabis therapeutics |
| cannabis therapy |
| cannabis treatment |
| cannabis-based medicine |
| cannabis-based pharmaceutical |
| cannabis-based pharmaceuticals |
| cannabis-based therapeutic |
| cannabis-based therapy |
| care tailored for people with disability |
| cbd health product |
| cbd health product developer |
| cbd infused health product |
| cbd medicine |
| cbd muscle rub |
| cbd pharma |
| cbd pharmaceutical |
| cbd pharmacy |
| cbd sleep solutions |
| cbd-based health product |
| cbt therapy |
| cbt-based program |
| celera drugs |
| cessation product |
| cessation system |
| cessation therapeutic |
| chemicals dependency therapy |
| chest pain |
| chest pain evaluation services |
| chest pain triage |
| child therapy |
| child therapy center |
| child therapy services |
| childhood adhd |
| childhood stress |
| children autism care |
| children behavioral health |
| children counseling |
| children mental care |
| children psychiatric hospital |
| children psychiatry |
| children therapeutic services |
| children's counseling |
| children's counseling center |
| chiropractic adjustments |
| chiropractic care |
| chiropractic care center |
| chiropractic care clinic |
| chiropractic care provider |
| chiropractic care service |
| chiropractic center |
| chiropractic clinic |
| chiropractic doctor |
| chiropractic healthcare |
| chiropractic massage |
| chiropractic service provider |
| chiropractic services |
| chiropractic therapy |
| chiropractic treatment |
| chiropractic x-ray |
| chiropractor |
| chiropractor clinic |
| chiropractor service |
| chiropractor treatment |
| chronic back pain |
| chronic back pain drugs |
| chronic back pain treatment |
| chronic inflammation therapeutics |
| chronic inflammation treatment |
| chronic inflammatory diseases |
| chronic inflammatory diseases treatment |
| chronic inflammatory disorders |
| chronic low back pain care |
| chronic nerve pain |
| chronic nerve pain device |
| chronic pain |
| chronic pain condition |
| chronic pain conditions |
| chronic pain control |
| chronic pain devices |
| chronic pain management |
| chronic pain management portal |
| chronic pain management service |
| chronic pain medication |
| chronic pain program |
| chronic pain reduction |
| chronic pain relief |
| chronic pain syndrome |
| chronic pain therapy |
| chronic pain treatment |
| chronic pain treatment services |
| chronic patient |
| chronic peripheral inflammation |
| chronic peripheral pain |
| chronic stress |
| chronic stress relief |
| cigarettes |
| cigarettes addiction recovery |
| cigarettes cigarettes |
| cigarettes manufacturer |
| cigarettes quit |
| cigarettes threat |
| cigarettes vape |
| claustrophobia treatment |
| clean medicinal cannabis |
| clinical behavioral services |
| clinical counselling |
| clinical hypnosis |
| clinical massage |
| clinical neurophsyiology |
| clinical pain |
| clinical psychologists |
| clinical psychology |
| clinical psychopharmacology |
| clinical therapeutics |
| clinical therapy |
| clinical therapy management |
| clinical therapy treatment |
| clinical trials in depression |
| cocaine addiction |
| cocaine addiction treatment |
| codeine phosphate |
| codeine tablets |
| cognition |
| cognition deficit |
| cognition enhancement |
| cognition impairment |
| cognition support |
| cognitive |
| cognitive alterations |
| cognitive analytics platform |
| cognitive assessment |
| cognitive assessment aids |
| cognitive assessment app |
| cognitive assessment service |
| cognitive behavior |
| cognitive behavior therapy |
| cognitive behavioral approach |
| cognitive behavioral platform |
| cognitive behavioral programs |
| cognitive behavioral therapist |
| cognitive behavioral therapy |
| cognitive behaviour analysis |
| cognitive behaviour therapy |
| cognitive behavioural therapy |
| cognitive care |
| cognitive care tools |
| cognitive changes |
| cognitive deficiencies |
| cognitive development |
| cognitive devices |
| cognitive difficulty |
| cognitive disease treatment |
| cognitive disorder |
| cognitive disorder detection |
| cognitive dysfunction |
| cognitive engagement |
| cognitive enhancer |
| cognitive function |
| cognitive function development |
| cognitive health |
| cognitive health assessment |
| cognitive health product |
| cognitive health rehabilitation |
| cognitive health screening |
| cognitive impairment |
| cognitive impairment care |
| cognitive impairment monitoring |
| cognitive impairment solution |
| cognitive impairment tracking |
| cognitive improvement |
| cognitive intervention |
| cognitive learning skills |
| cognitive measurement |
| cognitive milestone tracking |
| cognitive performance |
| cognitive processing therapy |
| cognitive psychophysics |
| cognitive rehabilitation |
| cognitive remediation |
| cognitive remediation platform |
| cognitive remediation tools |
| cognitive retraining |
| cognitive retraining service |
| cognitive science |
| cognitive screening test |
| cognitive search |
| cognitive skills |
| cognitive stimulation coaching |
| cognitive supplements |
| cognitive technology |
| cognitive test |
| cognitive testing |
| cognitive testing device |
| cognitive testing platform |
| cognitive therapeutic services |
| cognitive therapeutics |
| cognitive therapy |
| cognitive thinking skills |
| cognitive tools |
| cognitive training |
| cognitive training tools |
| cognitive wearable |
| cognitive-behavioral therapy treatment |
| cognitively-impaired patients |
| cold and heat therapy device |
| cold compression therapy product |
| cold laser |
| cold laser therapy services |
| cold packs |
| cold sleeves |
| cold therapy |
| cold therapy device |
| cold therapy equipment |
| cold therapy gel bags |
| cold therapy product |
| cold therapy provider |
| cold therapy system |
| cold therapy wraps |
| cold wraps manufacturer |
| combination therapy platform |
| communication therapy |
| community mental health |
| compassionate therapist |
| comprehensive psychiatric evaluation |
| comprehensive rehabilitation service |
| compulsive behavior |
| compulsive overeating |
| computer aided therapy |
| computer therapy |
| computerized psychological testing |
| consultation in pain management |
| control pain and infection |
| conventional therapy |
| conversation therapy |
| cooling therapy |
| corrective therapy |
| cortical stimulation |
| corticosteroid |
| counseling |
| counseling administration |
| counseling and therapy |
| counseling center |
| counseling clinic |
| counseling group |
| counseling needs |
| counseling pet |
| counseling platform |
| counseling practice |
| counseling program |
| counseling service |
| counseling service booking |
| counseling service platform |
| counseling service provider |
| counseling services |
| counseling sessions |
| counseling software |
| counseling therapy |
| counseling treatment |
| counselling |
| counselling center |
| counselling courses |
| counselling firm |
| counselling sessions |
| counselling software |
| counselling support |
| counselling treatment |
| counselor assistance |
| counter stimulation therapy |
| cranial electrotherapy stimulator |
| craniosacral therapy |
| crisis counselors |
| critical light treatment |
| cryo chamber |
| cryo shoulder |
| cryogenic therapy |
| cryotherapy |
| cryotherapy center |
| cryotherapy chamber |
| cryotherapy clinic |
| cryotherapy device |
| cryotherapy devices |
| cryotherapy equipment |
| cryotherapy equipment rental |
| cryotherapy machine |
| cryotherapy medical device |
| cryotherapy product |
| cryotherapy products |
| cryotherapy service |
| cryotherapy services |
| cryotherapy system |
| cryotherapy technology |
| cryotherapy therapy |
| cryotherapy treatment |
| cryothermic technology |
| cure back pain |
| customized therapy |
| cyryotherapy center |
| daily mood analyzing |
| ddiction treatment specialist |
| de-addiction program |
| de-addiction services |
| deep brain stimulation surgeries |
| deep brain stimulation surgery |
| deficit disorder |
| dementia and mental health clinic |
| dependency rehabilitation |
| depressed patients |
| depression |
| depression and anxiety |
| depression and anxiety treatment |
| depression app |
| depression care |
| depression care services |
| depression counseling |
| depression counselling |
| depression cure |
| depression diagnostics |
| depression drugs |
| depression management |
| depression management software |
| depression medication |
| depression overcome |
| depression problem solution |
| depression relief |
| depression solutions |
| depression technology |
| depression therapy |
| depression tracking app |
| depression treatment |
| depression treatment courses |
| depression treatment services |
| depressive |
| depressive disorder |
| depressive disorder drugs |
| deterrent patch technology |
| detox |
| detox care |
| detox center |
| detox planning |
| detox program |
| detox programmes |
| detox supplements |
| detox tablets |
| detox treatment |
| detoxification |
| detoxification and stabilization |
| detoxification center |
| detoxification product |
| detoxification program |
| development disorder treatment |
| developmental disabilities assistance |
| developmental disabilities intervention |
| developmental disabilities services |
| developmental disabilities treatment |
| developmental disorder therapy |
| developmental disorder training services |
| dexmedetomidine hcl |
| diagnose autism |
| dialectical behavior therapy |
| dialectical behaviour therapy |
| digital addiction recovery treatment |
| digital chronic pain management |
| digital cognitive assessment |
| digital cognitive behavioral therapy |
| digital counseling program |
| digital health therapy |
| digital mental health |
| digital mental health solutions |
| digital sleep therapy |
| digital smoking-cessation system |
| digital smoking-cessation systems |
| digital therapeutic |
| digital therapeutic device |
| digital therapeutic platform |
| digital therapeutic product |
| digital therapeutic services |
| digital therapeutic system |
| digital therapeutic tools |
| digital therapeutic treatment |
| digital therapeutics |
| digital therapeutics company |
| digital therapeutics firm |
| digital therapeutics for behavioral health |
| digital therapeutics platform |
| digital therapeutics platform developer |
| digital therapeutics software |
| digital therapist |
| digital therapy |
| digital therapy application |
| digital therapy interface |
| digital therapy platform |
| digitally-guided therapy |
| dipotassium tablets |
| disability aids |
| disability assessment |
| disability assistance care |
| disability care |
| disability care center |
| disability care giving |
| disability care home |
| disability care unit |
| disability claims |
| disability claims management |
| disability determination services |
| disability development care |
| disability device |
| disability discrimination |
| disability employment |
| disability equipment |
| disability evaluation |
| disability exam |
| disability examination service |
| disability health insurance |
| disability improvement training |
| disability insurance |
| disability insurance product |
| disability insurance service |
| disability management |
| disability management services |
| disability patients |
| disability product |
| disability program |
| disability research |
| disability service |
| disability services |
| disability support |
| disability support service |
| disability technology |
| disability therapy |
| disability tools |
| disability training |
| disability transport |
| disability transportation |
| disability treatment |
| disability treatment services |
| disability vehicles |
| disable care |
| disable care product |
| disable care services |
| disable habilitative service |
| disable people |
| disabled |
| disabled car |
| disabled care |
| disabled care and treatment |
| disabled care building |
| disabled care service |
| disabled home care |
| disabled person |
| disabled support |
| disbale care |
| disc pain therapy |
| disorder of sensory information processing |
| disorder therapy |
| dopamine transporters |
| drugs abuse |
| drugs abuse analysis |
| drugs abuse assessment tools |
| drugs abuse care |
| drugs abuse center |
| drugs abuse help center |
| drugs abuse prevention |
| drugs abuse testing services |
| drugs abuse treatment |
| drugs addict |
| drugs addiction |
| drugs addiction care |
| drugs addiction healing |
| drugs addiction rehab |
| drugs addiction solutions |
| drugs addiction therapy |
| drugs addiction therapy center |
| drugs addiction treatment |
| drugs addition |
| drugs analytics |
| drugs and alcohol |
| drugs and alcohol addiction |
| drugs and alcohol program |
| drugs and alcohol rehabilitation center |
| drugs and alcohol treatment |
| drugs detox |
| drugs detox center |
| drugs detox services |
| drugs detoxification |
| drugs disorder |
| drugs free pain relief |
| drugs intoxications |
| drugs of abuse |
| drugs overdose antidote |
| drugs overdose detection |
| drugs recovery |
| drugs rehab |
| drugs rehab center |
| drugs rehab device |
| drugs rehab facility |
| drugs rehab medicine |
| drugs rehabilitation |
| drugs rehabilitation center |
| drugs rehabilitation service |
| drugs rehabs |
| drugs related problems |
| drugs treatment programs |
| drugs usage solutions |
| drugs use disorder support |
| dysphasia |
| e cigarettes product |
| e-cig product |
| e-cigarette |
| e-cigarette product |
| e-cigarette seller |
| e-counselling |
| e-mental healthcare |
| early childhood intervention |
| early intensive behavioral intervention |
| early intervention program |
| early psychosis |
| eating disorder |
| eating disorder awareness |
| eating disorder care |
| eating disorder cure |
| eating disorder management |
| eating disorder program |
| eating disorder rehabilitation |
| eating disorder services |
| eating disorder solutions |
| eating disorder therapy |
| eating disorder treatment |
| eating disorder treatment services |
| eating syndrome |
| ecreation therapy |
| educational therapy |
| educational therapy services |
| elderly mental health |
| electric stimulation |
| electric stimulator |
| electric therapy |
| electrical muscle stimulation equipment |
| electrical nerve stimulation |
| electrical nerve stimulation machine |
| electrical stimulation |
| electrical stimulation device |
| electrical stimulation equipment |
| electrical stimulation technology |
| electrical stimulator therapy |
| electro neuro muscular therapy |
| electro-therapeutic device |
| electroanalgesic pain management |
| electroceutical device |
| electroconvulsive therapy |
| electronic cigarettes |
| electronic hot and cold band |
| electronic media therapy |
| electronic nerve stimulator |
| electronic nicotine |
| electrostimulation device |
| electrostimulation therapy |
| electrostimulation wearable |
| electrotherapeutic |
| electrotherapeutic apparatus |
| electrotherapeutic apparatus manufacturing |
| electrotherapeutic devices |
| electrotherapy |
| electrotherapy device |
| electrotherapy device manufacturer |
| electrotherapy product |
| emergency psychiatric care |
| emergency psychiatric treatment |
| emotional assessment tools |
| emotional care |
| emotional care services |
| emotional child care |
| emotional counseling |
| emotional development |
| emotional developmental need |
| emotional disorder |
| emotional health |
| emotional health assessment |
| emotional health assistant |
| emotional health care |
| emotional health intervention |
| emotional health platform |
| emotional health treatment |
| emotional issues |
| emotional learning |
| emotional learning program |
| emotional physiology |
| emotional problem |
| emotional robots |
| emotional support |
| emotional support animal |
| emotional support systems |
| emotional trauma medicine |
| emotional treatment |
| emotional wellbeing |
| emotional wellness |
| emotions analytics |
| emotions tracking platform |
| employee mental health |
| endocannabinoid |
| endocannabinoid system |
| evidence-based therapy |
| executive functioning |
| exposure therapy |
| facet joint reconstruction |
| facet joint syndrome |
| facet syndrome |
| family counseling service |
| family counseling services |
| family therapy |
| family therapy center |
| fast-acting relief |
| fentanyl addiction vaccines |
| fentanyl detection |
| fibromyalgia |
| fibromyalgia syndrome |
| fibromyalgia treatment |
| fight anxiety |
| fight depression |
| focal pain therapy |
| foot and ankle pain |
| foot pain |
| frozen shoulder therapy |
| gabapentin |
| generalized anxiety disorder |
| geriatric psychiatry |
| geriatric therapy |
| gero-psychiatric treatment |
| geropsychiatric hospital unit |
| geropsychiatric treatment |
| geropsychiatry |
| geropsychology |
| golf therapy services |
| gout |
| group counseling |
| group counselling |
| group psychiatry |
| group therapy |
| group therapy facilitation |
| group therapy platform |
| hallucinations treatment |
| hallucinogenic drugs |
| headach treatment |
| headache care |
| headache disorder |
| headache drugs |
| headache management service |
| headache medicine |
| headache relief |
| headache therapy |
| headache treatment |
| headaches |
| headaches and migraine |
| headaches treatment |
| healing light |
| health behavior analysis |
| health behavior change |
| health cannabis |
| health care therapy |
| healthcare behavioral research |
| healthcare counseling platform |
| healthcare counseling services |
| healthcare dispensary |
| healthcare marijuana product |
| heat pack manufacturer |
| heat therapy |
| heel pain |
| heel pain care |
| heel pain treatment |
| hemp based medicine |
| herniated pain |
| heroin addiction |
| heroin addiction care |
| heroin detox |
| herpetic neuralgia |
| herpetic neuralgia treatment |
| hip pain |
| hoarding disorder management |
| holistic mental health |
| holistic therapy |
| home and behavioral health care |
| home health therapy |
| home-based addiction treatment |
| home-based therapy |
| hot and cold therapy |
| hot wraps |
| human behavior |
| human behavior analysis |
| human cognition |
| human memory disorder |
| hydrocodone bitartrate |
| hydrotherapy equipment |
| hyperactivity disorder |
| hyperactivity disorder treatment |
| i/dd agency management software |
| ibogaine treatment |
| ibuprofen |
| ice compression |
| ice therapy system |
| icepack therapy |
| idd service provider |
| implantables neuromodulation device |
| implantables neuromodulation technologies |
| implantables neurostimulator |
| implantables simulation |
| implantables stimulation device |
| implantables stimulation devices |
| improving mental health |
| in home addiction treatment |
| in home family support |
| in home rehabilitation |
| in home therapy |
| in home therapy services |
| in house therapy |
| in-house therapy |
| in-person therapy |
| independent adult care services |
| individual counseling |
| individual psychotherapy |
| individual therapy |
| individual therapy sessions |
| individualized drug testing for pain management |
| individualized mental health |
| individualized therapy |
| individuals with developmental disabilities |
| inflammation |
| inflammation care |
| inflammation control |
| inflammation cure |
| inflammation drugs |
| inflammation lozenge |
| inflammation management |
| inflammation medication |
| inflammation monitoring |
| inflammation monitoring device |
| inflammation product |
| inflammation therapeutics |
| inflammation therapy |
| inflammation treatment |
| inflammation-based disease |
| inflammation-focused |
| inflammatory care |
| inflammatory care drugs |
| inflammatory disease |
| inflammatory disease care |
| inflammatory disease drugs |
| inflammatory drug |
| inflammatory mediator |
| inflammatory medicine |
| inflammatory musculoskeletal |
| inflammatory pain |
| inflammatory pain treatment |
| inflammatory pathway |
| inflammatory process |
| inflammatory therapy |
| inflammatory treatment |
| inflammatory-based disease |
| infrared therapy |
| inhalants |
| inhaled drugs |
| injury and pain |
| inpatient behavioral treatment |
| inpatient mental health |
| inpatient psychiatric |
| inpatient psychiatric care |
| inpatient psychiatric clinic |
| inpatient psychiatric hospital |
| inpatient psychiatric services |
| inpatient psychiatric treatment |
| inpatient rehab facility |
| inpatient rehabilitation |
| inpatient rehabilitation center |
| inpatient rehabilitation facility |
| inpatient rehabilitation hospital |
| inpatient rehabilitation services |
| inpatient substance |
| insomnia |
| insomnia care |
| insomnia cure |
| insomnia medicine |
| insomnia therapy |
| insomnia therapy device |
| insomnia treatment |
| insomnia treatment services |
| integrated behavioral health |
| integrated behavioral science |
| integrated pain management |
| integrated therapy |
| integrative harm reduction |
| intellectual and developmental disabilities services |
| intellectual disability |
| intellectual disability care |
| intellectual disability healthcare |
| intellectual disability services |
| intellectual disorder |
| intellectual healthcare |
| intensive behavioral intervention |
| intensive care therapy |
| intensive home-based counseling |
| intensive in-person behavioral therapy |
| intensive outpatient program |
| intensive outpatient programming |
| interactive mental health |
| interventional pain |
| interventional pain management |
| interventional pain management solutions |
| intractable pain |
| jaw pain |
| joint pain |
| joint pain medicine |
| joint pain relief |
| joint pain tracking |
| joint pain treatment |
| ketamine |
| ketamine clinic |
| ketamine delivery |
| ketamine delivery system |
| ketamine drugs |
| ketamine infusion clinic |
| ketamine infusion therapy |
| ketamine psychotherapy |
| ketamine therapy |
| ketamine treatment |
| kids counseling |
| knee pain |
| knee pain product |
| knee pain relief |
| knee pain solution |
| knee pain therapy |
| kratom capsules |
| labor pain |
| laser for pain |
| laser therapy |
| laser treatment |
| laser treatment service |
| legging pain |
| legging pain treatment |
| licensed addictionologist |
| licensed counseling |
| licensed social worker |
| licensed therapists |
| lidocaine |
| life-changing therapy |
| life-threatening drugs |
| light therapy |
| light therapy device |
| light therapy product |
| light therapy solution |
| live in rehabilitation |
| live meditation platform |
| live meditation service |
| local analgesia |
| local inflammatory response |
| long term counseling |
| low back pain |
| low back pain cure |
| low level light therapy |
| lower back pain |
| lower back pain reduce |
| lower back pain relief |
| lower back pain treatment |
| lsd |
| magnetic brain stimulation |
| magnetic nerve stimulator |
| magnetic stimulation |
| magnetic stimulation device |
| maintenance drugs recipient |
| major depression |
| major depressive disorder treatment drug |
| major depressive disorders treatment drug |
| maladaptive behavior |
| managing mental health |
| manuals physical therapy |
| manuals therapist |
| manuals therapy |
| manuals therapy techniques |
| marijuana healing |
| marijuana medicine |
| marijuana medicine developer |
| marijuana therapy |
| marijuana therapy education |
| marijuana therapy education provider |
| marijuana therapy services |
| marijuana therapy services provider |
| marijuana treatment |
| massage service provider |
| massage services |
| massage therapists |
| massage therapists information |
| massage therapy |
| massage therapy center |
| massage therapy firm |
| massage therapy provider |
| massage therapy services |
| massage therepy |
| massage treatment |
| maternal mental |
| maternal mental health |
| mdd drug development |
| mdd management |
| mdd management platform |
| mdd treatment drugs |
| mdma therapy |
| meantal health care |
| measuring stress leveling |
| medical acupuncture |
| medical cannabi |
| medical cannabidiol |
| medical cannabinoid |
| medical cannabis |
| medical cannabis businesses |
| medical cannabis company |
| medical cannabis cultivation |
| medical cannabis industry |
| medical cannabis producer |
| medical cannabis product |
| medical cannabis project |
| medical cannabis treatment |
| medical detoxification |
| medical disability management |
| medical games therapy software |
| medical marijuana |
| medical marijuana clinic |
| medical marijuana company |
| medical marijuana cultivation |
| medical marijuana dispensary |
| medical marijuana treatment |
| medical rehab |
| medically assisted treatment |
| medication abuse |
| medication-assisted recovery services |
| medication-assisted treatment |
| medicine addiction |
| meditation therapy |
| menstrual pain |
| menstrual pain relief product |
| menstrual pain treatment |
| menstrual relief |
| mental ailments |
| mental and behavioral health services |
| mental assessment |
| mental assistance |
| mental care |
| mental care assistance |
| mental care center |
| mental care clinic |
| mental care facilities |
| mental care platform |
| mental care services |
| mental care software |
| mental care tools |
| mental care treatment |
| mental clinic |
| mental consultation |
| mental counseling |
| mental counselling |
| mental crisis situation |
| mental crisis stabilization |
| mental depression |
| mental depression treatment |
| mental disability |
| mental disability care |
| mental disorder |
| mental disorder app |
| mental disorder care |
| mental disorder consultant |
| mental disorder treatment |
| mental distress treatment |
| mental facility |
| mental health |
| mental health advancement services |
| mental health advocacy |
| mental health agency |
| mental health analysis |
| mental health analytics |
| mental health app |
| mental health application |
| mental health assessment |
| mental health assessment platform |
| mental health assessment software |
| mental health assistance |
| mental health awareness |
| mental health benefits |
| mental health betterment |
| mental health books |
| mental health campaign |
| mental health care |
| mental health care center |
| mental health care housing facility |
| mental health care medication |
| mental health care platform |
| mental health care provider |
| mental health care service |
| mental health care therapists |
| mental health center |
| mental health chatbot |
| mental health claim |
| mental health clinic |
| mental health clinic operator |
| mental health coaches app |
| mental health coaching |
| mental health community |
| mental health company |
| mental health concerns |
| mental health conditions |
| mental health consultancy platform |
| mental health counseling |
| mental health crisis solution |
| mental health cure |
| mental health delivery |
| mental health diagnostic system |
| mental health diagnostic technology |
| mental health diagnostics |
| mental health directories |
| mental health disorder |
| mental health disorder treatment |
| mental health disparities |
| mental health education service |
| mental health evaluation |
| mental health expertise |
| mental health facility |
| mental health firm |
| mental health friendly workplace |
| mental health home |
| mental health improvement |
| mental health improvement app |
| mental health information |
| mental health insights |
| mental health issues |
| mental health issues care |
| mental health issues treatment |
| mental health management |
| mental health management software |
| mental health medicine |
| mental health medicine maker |
| mental health monitoring |
| mental health monitoring application |
| mental health monitoring device |
| mental health monitoring technology |
| mental health needs |
| mental health outcomes |
| mental health outcomes software |
| mental health platform |
| mental health practices |
| mental health practitioner |
| mental health problem treatment |
| mental health professionals |
| mental health professionals database |
| mental health program |
| mental health providers |
| mental health record |
| mental health record platform |
| mental health recording software |
| mental health recovery |
| mental health rehabilitation |
| mental health reporting |
| mental health research |
| mental health screening |
| mental health screening tools |
| mental health seekers |
| mental health service provider |
| mental health services |
| mental health services platform |
| mental health services provider |
| mental health sessions |
| mental health software |
| mental health solutions |
| mental health stability |
| mental health status tracking |
| mental health struggles |
| mental health supplement |
| mental health support |
| mental health support services |
| mental health system |
| mental health tech |
| mental health technology |
| mental health therapeutic |
| mental health therapists |
| mental health therapy |
| mental health tool |
| mental health tracker |
| mental health tracking |
| mental health tracking application |
| mental health tracking platform |
| mental health training |
| mental health treatment |
| mental health treatment center |
| mental health treatment digitally |
| mental health treatment facility |
| mental health treatment provider |
| mental health treatment service |
| mental health wellbeing |
| mental health workshops |
| mental healthcar |
| mental healthcare |
| mental healthcare app |
| mental healthcare application |
| mental healthcare center |
| mental healthcare clinic |
| mental healthcare company |
| mental healthcare counseling |
| mental healthcare experts |
| mental healthcare hospital |
| mental healthcare monitoring |
| mental healthcare platform |
| mental healthcare service |
| mental healthcare software |
| mental healthcare solutions |
| mental healthcare system |
| mental healthcare technology |
| mental healthcare therapy |
| mental healthcare tools |
| mental healthcare treatment |
| mental healthcare unit |
| mental help |
| mental hospital |
| mental illness |
| mental illness care |
| mental illness diagnostics |
| mental illness healthcare |
| mental illness software |
| mental illness solutions |
| mental illness system |
| mental illness test |
| mental illness therapy |
| mental illness treatment |
| mental illness treatment company |
| mental impairment |
| mental pathology |
| mental patient |
| mental patient therapy |
| mental patient-care |
| mental problems |
| mental services |
| mental state analysis |
| mental stimulation |
| mental stimulation platform |
| mental stress prevention |
| mental support service |
| mental therapeutics |
| mental therapist platform |
| mental therapists appointment |
| mental therapy |
| mental therapy program |
| mental therapy services |
| mental treatment |
| mental treatment app |
| mental treatment facility |
| mental treatment services |
| mental-health services |
| mental-health treatment |
| mental-health treatment center |
| mentally challenged |
| mentally disabled |
| mentally disabled care |
| mentally ill patients therapy |
| meth addiction |
| meth detox treatment |
| methadone |
| methadone drugs |
| methadone maintenance |
| methadone maintenance treatment |
| methadone treatment |
| methadone treatment center |
| methamphetamine |
| methamphetamine dependence |
| methamphetamine detection |
| mhealth |
| mhealth app |
| mhealth application |
| mhealth platform |
| mhealth services |
| mhealth-mobile health |
| microdosed therapeutics |
| microdosing |
| microwave heat pack |
| microwave heat pack manufacturer |
| migraine headache relief |
| migraine pain |
| migraine relief device |
| migraine relief services |
| migraine-relief |
| mild cognitive impairment |
| minor pain |
| mirtazapine product |
| misuse prevention software |
| mobile mental health platform |
| mobile therapy |
| mobile therapy platform |
| moderate pain relief |
| monitoring therapy |
| mood analysis |
| mood and anxiety disorders treatment |
| mood disorders drugs |
| mood disorders treatment |
| mood identifier |
| mood management |
| mood remedy |
| mood tracking application |
| morphine replacement |
| motivational therapy |
| multi sensory therapy |
| multi-sensory stimulation |
| multi-sensory therapy |
| multiple behavior health |
| multisensory stimulation |
| muscle pain |
| muscle pain care |
| muscle pain lotion |
| muscle pain relief |
| muscle pain therapeutic device |
| muscle pain treatment |
| muscle soreness |
| muscle stimulation |
| muscle stimulation device |
| muscle stimulation equipment |
| muscle stimulation system |
| muscle stimulator |
| muscular pain relief |
| musculoskeletal injuries pain |
| musculoskeletal pain |
| musculoskeletal pain drugs |
| musculoskeletal pain treatment |
| music therapeutic effect |
| music therapists online |
| music therapy |
| music therapy app |
| music therapy services |
| musicals therapy |
| naloxone hydrochloride product |
| narcotic |
| narcotic detection |
| narcotic drugs |
| narcotic screening |
| narcotic treatment |
| narcotics |
| narcotics breathalyzer |
| narcotics cabinets |
| narcotics control |
| narcotics dependent treatment |
| narcotics management |
| narcotics testing |
| nascent behavioral health sector |
| natural pain management |
| natural stimulation |
| neck pain |
| neck pain relief |
| neck pain treatment |
| neck pain treatment device |
| neck pains |
| negative pressure therapy |
| nerve pain |
| nerve pain alleviation treatment |
| nerve pain relief |
| nerve pain treatment |
| nerve signalling disorder treatment |
| nerve stimulation |
| nerve stimulation device |
| nerve stimulation product |
| nerve stimulation system |
| nerve stimulation therapy |
| nerve stimulation therapy platform |
| nerve stimulator |
| neural stimulation |
| neural stimulation system |
| neural stimulation treatment |
| neuro pain |
| neurobehavioral |
| neurobehavioral disorder treatment |
| neurobehavioral evaluation |
| neurobehavioral rehabilitation |
| neurobehavioral test |
| neurological pain |
| neurological rehabilitation |
| neurology pain management |
| neuromodulation platform |
| neuromodulation research |
| neuromodulation system |
| neuromodulation techniques |
| neuromodulation technologies |
| neuromodulation technologies developer |
| neuromodulation therapy device |
| neuromodulation tools |
| neuromodulation treatment |
| neuromuscular device |
| neuron stimulation |
| neuropathic analgesic |
| neuropathic disorder therapy |
| neuropathic pain |
| neuropathic pain drugs |
| neuropathic pain product |
| neuropathic pain treatment |
| neuropathic painkiller |
| neuropathic treatment |
| neuropathy |
| neuropathy therapy |
| neuropathy treatment |
| neuropsychiatric |
| neuropsychiatric disease |
| neuropsychiatric disease treatment |
| neuropsychiatric disorder |
| neuropsychiatric disorder drugs |
| neuropsychiatric disorder treatment |
| neuropsychiatric healthcare services |
| neuropsychiatric therapy |
| neuropsychiatric treatment |
| neuropsychiatry |
| neuropsychiatry drugs |
| neuropsychological analysis |
| neuropsychological assessment |
| neuropsychological evaluation |
| neuropsychological testing |
| neuropsychology |
| neuropsychology research |
| neurostimulation |
| neurostimulation device |
| neurostimulation medical device |
| neurostimulation products |
| neurostimulation system |
| neurostimulation technology |
| neurostimulation technology device |
| neurostimulation therapy |
| neurostimulation wearable device |
| neurostimulation wearables |
| neurostimulator |
| neurostimulator electrode |
| neurostimulator manufacturer |
| neurotherapeutics |
| neurotherapeutics development |
| neurotherapeutics platform |
| neurotherapist locator |
| neurotherapy |
| nicotine |
| nicotine addiction |
| nicotine addiction treatment |
| nicotine aerosol |
| nicotine alternative |
| nicotine chewing gum |
| nicotine conjugate vaccine |
| nicotine consumption |
| nicotine consumption control |
| nicotine consumption reduction |
| nicotine de-addiction |
| nicotine free |
| nicotine gum |
| nicotine gum product |
| nicotine intake detection |
| nicotine intake tracking |
| nicotine items |
| nicotine management |
| nicotine pouches |
| nicotine product |
| nicotine reduction |
| nicotine supplements |
| nicotine vape |
| nicotine vaporizer |
| nicotinic drugs |
| nicotinic receptor |
| nicotinic receptor drugs |
| nitrous oxide |
| nitrous oxide dispenser |
| non addictive drugs |
| non alcohol beverage |
| non narcotic treatment |
| non-addictive drugs |
| non-addictive pain management |
| non-addictive pain treatment |
| non-addictive painkiller |
| non-alcoholic ginger drinks |
| non-electric heat pack |
| non-habit-forming painkiller |
| non-narcotic analgesic |
| non-narcotic drugs |
| non-narcotic respiratory drug |
| non-opiate drugs |
| non-opioid |
| non-opioid drugs |
| non-opioid molecules spray |
| non-opioid pain management |
| non-opioid pain therapeutics |
| non-opioid pain therapy |
| non-opioid product |
| non-opioid therapeutic |
| non-opioid therapeutics |
| non-opioid therapy |
| non-pharmacological pain treatment |
| noninvasive nerve stimulator |
| noninvasive neuromodulation therapy |
| noninvasive neuromodulation treatment |
| noninvasive neurostimulation |
| noninvasive stimulator |
| novel non-opioid medication |
| nursing therapy |
| ocd disorder |
| ocd treatment |
| offers detox program |
| offers therapy |
| on demand teletherapy platform |
| on demand therapist |
| on demand therapy |
| on-call therapy |
| online addiction support |
| online adhd management |
| online adhd training |
| online adhd treatment |
| online cognitive screening test |
| online counseling |
| online counseling platform |
| online counseling service |
| online counseling session |
| online counselling |
| online depression courses |
| online depression therapy |
| online health therapist |
| online health therapy |
| online mental health |
| online mental health assessment |
| online mental health coaching |
| online mental health sessions |
| online mental healthcare |
| online mental therapy |
| online pain management |
| online rehab facility |
| online therapeutic meeting |
| online therapeutic services |
| online therapist |
| online therapist directory |
| online therapy |
| online therapy platform |
| online therapy program |
| online therapy services |
| online therapy solutions |
| onsite psychiatric |
| onsite rehab |
| onsite rehabilitation |
| onsite therapy |
| opiate addiction |
| opiate addiction treatment |
| opiate alternative |
| opiate detoxification center |
| opiate treatment |
| opioid |
| opioid abuse |
| opioid addiction |
| opioid addiction center |
| opioid addiction clinic |
| opioid addiction curing services |
| opioid addiction program |
| opioid addiction recovery |
| opioid addiction treatment |
| opioid agonist |
| opioid alternative |
| opioid analgesic |
| opioid antidote |
| opioid care |
| opioid care program |
| opioid care treatment clinic |
| opioid crisis |
| opioid dependence |
| opioid dependence treatment |
| opioid dependencies |
| opioid detection |
| opioid disorder recovery |
| opioid drugs addicts |
| opioid epidemic |
| opioid epidemics |
| opioid formulation |
| opioid inhaler |
| opioid metabolites detection |
| opioid overdose |
| opioid overdose drugs |
| opioid overdose treatment |
| opioid pain |
| opioid pain drugs |
| opioid pain management |
| opioid pain therapy |
| opioid prescription |
| opioid recovery bracelets |
| opioid substitute |
| opioid substitution |
| opioid therapy |
| opioid treatment |
| opioid treatment center |
| opioid treatment clinic |
| opioid treatment programs |
| opioid treatment services |
| opioid use disorder |
| opioid use prevention |
| opioid withdrawal |
| opioid-free anti-pain therapy |
| opioids |
| opioids medicine |
| opium-based control substances |
| oral analgesic |
| oral analgesic tablets |
| oral anti-inflammatory therapy |
| oral dosing pain management |
| oral pain medication |
| orally active analgesic drug |
| organic medicinal cannabis licenses |
| organic medicinal marijuana |
| organic muscle relaxer |
| orthopedic pain |
| orthopedic pain treatment |
| outpatient addiction |
| outpatient addiction recovery |
| outpatient addiction treatment |
| outpatient behavioral health |
| outpatient behavioral platform |
| outpatient behavioral treatment |
| outpatient counseling |
| outpatient counseling service |
| outpatient counseling services |
| outpatient detox care |
| outpatient detox services |
| outpatient detoxification |
| outpatient medication therapy |
| outpatient medication-assisted treatment |
| outpatient mental health |
| outpatient mental health clinic |
| outpatient mental health organization |
| outpatient mental health platform |
| outpatient mental health services |
| outpatient psychiatric care |
| outpatient psychiatric services |
| outpatient psychiatry |
| outpatient recovery program |
| outpatient recovery services |
| outpatient rehab |
| outpatient rehabilitation clinic |
| outpatient rehabilitation provider |
| outpatient rehabilitation service |
| outpatient rehabilitation therapy |
| outpatient substance abuse treatment |
| outpatient therapy |
| outpatient therapy center |
| outpatient therapy clinic |
| outpatient therapy device |
| outpatient therapy services |
| outpatient treatment |
| outpatient treatment center |
| outpatient treatment services |
| outsourced therapy |
| outsourced therapy management |
| outsourced therapy provider |
| overcome addiction |
| overdose drugs |
| overdose protection |
| oxycodone |
| oxycodone hydrochloride tablets |
| pain |
| pain and cancer |
| pain and emotional support |
| pain assessment |
| pain assessment application |
| pain assessment device |
| pain assessment system |
| pain associated disease |
| pain balm |
| pain blocks |
| pain care |
| pain care device |
| pain care management |
| pain care program |
| pain care therapy |
| pain care treatment |
| pain center |
| pain clinic |
| pain clinicians |
| pain conditions |
| pain control |
| pain control clinic |
| pain control specialist |
| pain control system |
| pain correcting device |
| pain cream |
| pain cure |
| pain curing services |
| pain diagnostics |
| pain disorder |
| pain drugs |
| pain healing drugs |
| pain healing medicine |
| pain healing ointments |
| pain injection |
| pain killer |
| pain killer alternative |
| pain killer drugs |
| pain killer spray |
| pain manageme |
| pain management |
| pain management care |
| pain management center |
| pain management clinic |
| pain management device |
| pain management drug |
| pain management events |
| pain management injection |
| pain management medicine |
| pain management physician |
| pain management practices |
| pain management product |
| pain management program |
| pain management service |
| pain management software |
| pain management solutions |
| pain management surgeries |
| pain management surgery |
| pain management system |
| pain management technology |
| pain management testing |
| pain management therapy |
| pain management tool |
| pain management treatment |
| pain measurement |
| pain measurement device |
| pain measurement platform |
| pain medication |
| pain medication monitoring |
| pain medicine |
| pain mitigation |
| pain modulation |
| pain monitor |
| pain monitoring |
| pain monitoring device |
| pain monitoring software |
| pain monitoring system |
| pain physician |
| pain pills addiction |
| pain prevention |
| pain product |
| pain program |
| pain psychology |
| pain pumps medication |
| pain recovery |
| pain reducing device |
| pain reducing medication |
| pain reduction |
| pain reduction treatment |
| pain rehabilitation |
| pain related product |
| pain relief |
| pain relief alternative |
| pain relief application |
| pain relief center |
| pain relief clinic |
| pain relief cream |
| pain relief devices |
| pain relief drugs |
| pain relief formula |
| pain relief garments |
| pain relief gel |
| pain relief lotion |
| pain relief management |
| pain relief medicine |
| pain relief oil |
| pain relief product |
| pain relief product manufacturer |
| pain relief property |
| pain relief spray |
| pain relief system |
| pain relief technology |
| pain relief therapeutics |
| pain relief therapy |
| pain relief training |
| pain relief treatment |
| pain relief wearables |
| pain reliever |
| pain reliever drugs |
| pain reliever products |
| pain reliever spray |
| pain relieving cream |
| pain relieving machine |
| pain relieving medication |
| pain service |
| pain signal transmission |
| pain signaling channel blocker |
| pain solution |
| pain specialist |
| pain specialty clinic |
| pain symptoms |
| pain syndrome |
| pain test |
| pain therapeutic |
| pain therapeutics |
| pain therapy |
| pain therapy systems |
| pain therapy treatment |
| pain tracking |
| pain tracking application |
| pain treatment |
| pain treatment care |
| pain treatment center |
| pain treatment device |
| pain treatment services |
| pain treatment therapeutics |
| pain treatment therapy |
| painkiller |
| painkiller development |
| painkiller discovery |
| painkiller maker |
| paracetamol |
| parental therapy |
| patient therapy |
| patient therapy compliance |
| patient therapy improvement |
| patient-specific therapy |
| pbm therapy |
| pediatric autism |
| pediatric behavioral |
| pediatric behavioral health |
| pediatric mental health |
| pediatric therapeutic listening |
| pediatric therapy |
| pediatric therapy application |
| pediatric therapy center |
| pediatric therapy company |
| pediatric therapy services |
| peer counseling service provider |
| peer counseling software |
| peer counseling tools |
| peer support |
| peer support network |
| peer therapy software |
| peer therapy tools |
| pelvic pain treatment services |
| pennsaid drugs |
| peptides agonist |
| period pain |
| period pain treatment |
| period relief |
| peripheral neuropathic pain |
| peripheral neuropathy |
| personality disorder treatment |
| personalized cannabis treatment |
| personalized crisis safety |
| personalized psychiatric care |
| personalized recovery |
| personalized recovery planning |
| personalized rehabilitation |
| personalized therapists |
| personalized therapy |
| personalized therapy services |
| personalized therapy tools |
| personalized virtual reality therapy |
| phobia |
| phobia treatment |
| photobiomodulation |
| photobiomodulation devices |
| photobiomodulation product |
| photobiomodulation technology |
| photobiomodulation therapy |
| phototherapy |
| phototherapy equipment |
| phototherapy lighting systems |
| phototherapy research |
| phototherapy system |
| phototherapy technology |
| physical pain treatment |
| pills addiction treatment |
| piroxicam capsules |
| positive psychology |
| post traumatic distress syndrome |
| post-procedural pain |
| post-surgical pain and limitations |
| post-traumatic stress |
| post-traumatic stress disorder treatment |
| post-traumatic stress treatment |
| postherpetic neuralgia |
| postoperative pain |
| postoperative pain management |
| postoperative pain treatment |
| posttraumatic stress disorder |
| prescription drugs abuse |
| prescription drugs abuse prevention |
| prescription drugs monitoring |
| prescription narcotics |
| prescription pain medication |
| prescription therapeutics |
| prescription therapy |
| pressure ulcers relief |
| private rehab facility |
| private rehab operator |
| private therapeutic school |
| private therapy services |
| procaine |
| propranolol hydrochloride |
| propranolol pharmaceuticals |
| prospira paincare treatment |
| providing therapy |
| psilocybin |
| psilocybin derivatives |
| psilocybin farming |
| psilocybin medicine |
| psilocybin mushrooms |
| psilocybin mushrooms products |
| psilocybin products |
| psilocybin research |
| psilocybin therapy |
| psilocybin therapy provider |
| psychedelic compound |
| psychedelic drug developer |
| psychedelic drug therapy |
| psychedelic drugs |
| psychedelic healthcare |
| psychedelic medicine |
| psychedelic model |
| psychedelic mushrooms |
| psychedelic pharmaceutical medicine |
| psychedelic product |
| psychedelic research |
| psychedelic science |
| psychedelic startup |
| psychedelic teletherapy |
| psychedelic teletherapy platform |
| psychedelic therapeutics |
| psychedelic therapy |
| psychedelic therapy application |
| psychedelic treatment |
| psychedelic-assisted therapy |
| psychedelic-derived drugs |
| psychedelic-derived medicine |
| psychedelics |
| psychedelics drugs |
| psychedelics industry |
| psychedelics therapy |
| psychedelics treatment |
| psychiatric |
| psychiatric & behavioral services |
| psychiatric assessment |
| psychiatric beds |
| psychiatric care |
| psychiatric care center |
| psychiatric center |
| psychiatric clinic |
| psychiatric condition |
| psychiatric consultation |
| psychiatric counseling |
| psychiatric counseling services |
| psychiatric cure |
| psychiatric diagnostics |
| psychiatric disease |
| psychiatric disease drugs |
| psychiatric disease treatment |
| psychiatric disorder |
| psychiatric disorder solution |
| psychiatric disorder treatment |
| psychiatric disorder treatment facility |
| psychiatric drugs |
| psychiatric emergency |
| psychiatric evaluation |
| psychiatric facility |
| psychiatric health care facility |
| psychiatric health treatment |
| psychiatric healthcare |
| psychiatric healthcare service |
| psychiatric help |
| psychiatric hospital |
| psychiatric illness |
| psychiatric illness treatment |
| psychiatric inpatient |
| psychiatric inpatient services |
| psychiatric inpatient treatment |
| psychiatric management |
| psychiatric medication |
| psychiatric medicine |
| psychiatric nursing |
| psychiatric nursing care |
| psychiatric observation |
| psychiatric oversight |
| psychiatric problem |
| psychiatric providers |
| psychiatric rehabilitation |
| psychiatric rehabilitation service |
| psychiatric residential treatment |
| psychiatric services |
| psychiatric services firm |
| psychiatric specialties |
| psychiatric stabilization |
| psychiatric study |
| psychiatric support |
| psychiatric therapy |
| psychiatric treatment |
| psychiatric treatment center |
| psychiatric treatment facility |
| psychiatric treatment provider |
| psychiatric treatment services |
| psychiatrist |
| psychiatrist and mental health |
| psychiatrist center |
| psychiatrist healthcare |
| psychiatrist meeting |
| psychiatry |
| psychiatry & counseling |
| psychiatry and psychotherapy services |
| psychiatry care services |
| psychiatry clinic |
| psychiatry diagnostics |
| psychiatry disease treatment |
| psychiatry equipmenet |
| psychiatry service |
| psychiatry treatment |
| psycho-social counseling |
| psycho-therapeutic treatment |
| psychoactive cannabinoid |
| psychoactive drugs |
| psychoactive effects |
| psychoactive therapy services |
| psychoactivity drugs |
| psychological |
| psychological and psychiatric services |
| psychological assessment |
| psychological assessment application |
| psychological assessment platform |
| psychological assessment tools |
| psychological care |
| psychological care application |
| psychological care services |
| psychological center |
| psychological clinic |
| psychological conditions |
| psychological counseling |
| psychological counseling platform |
| psychological counseling robots |
| psychological counseling service |
| psychological counseling services |
| psychological counselor |
| psychological data |
| psychological evaluation |
| psychological guidance |
| psychological health |
| psychological help |
| psychological problems |
| psychological reliance services |
| psychological screening |
| psychological service provider |
| psychological services |
| psychological services provider |
| psychological study |
| psychological support |
| psychological symptoms |
| psychological techniques |
| psychological test |
| psychological testing |
| psychological testing services |
| psychological therapy |
| psychological therapy using virtual reality |
| psychological tools |
| psychological treatment |
| psychological understanding |
| psychologist |
| psychologist consultation |
| psychology |
| psychology analysis |
| psychology assessment |
| psychology center |
| psychology consulting |
| psychology doctor |
| psychology service platform |
| psychology services |
| psychology therapy |
| psychology tools |
| psychology treatment |
| psychology tutorials |
| psychopathology |
| psychopathology drugs |
| psychopathology treatment |
| psychopharmacology |
| psychosis treatment |
| psychosocial |
| psychosocial care |
| psychosocial disorder treatment |
| psychosocial services |
| psychosocial support |
| psychosocial support services |
| psychosocial supportive service |
| psychosocial treatment |
| psychotherapeutic care services |
| psychotherapeutic treatment |
| psychotherapists-patients |
| psychotherapy |
| psychotherapy care |
| psychotherapy counseling |
| psychotherapy evaluation |
| psychotherapy platform |
| psychotherapy services |
| psychotherapy services provider |
| psychotherapy treatment |
| psychotic disorder |
| psychotropic medication |
| psycotherapy practioners |
| PsyD |
| ptsd |
| ptsd care |
| ptsd cure |
| ptsd evaluation |
| ptsd treatment |
| quit cigarettes |
| quit smoking |
| quit smoking device |
| recovery care |
| recovery center |
| recovery drugs |
| recovery therapy |
| recovery tools |
| recovery treatment |
| recovery-tracking app |
| recreational therapy |
| red light |
| red light therapy |
| red light therapy device |
| reduce drugs consumption |
| reduce pain and stress |
| rehab |
| rehab & therapy |
| rehab business |
| rehab care |
| rehab center |
| rehab clinic |
| rehab department |
| rehab facilities |
| rehab facility |
| rehab facility care |
| rehab programs |
| rehab programs provider |
| rehab protocol |
| rehab service provider |
| rehab services |
| rehab services provider |
| rehab therapists |
| rehabiliation center |
| rehabilitaion facility |
| rehabilitation |
| rehabilitation agency |
| rehabilitation assessment service |
| rehabilitation care facilities |
| rehabilitation care services |
| rehabilitation center |
| rehabilitation center operator |
| rehabilitation centres |
| rehabilitation clinic |
| rehabilitation clinic services |
| rehabilitation facility |
| rehabilitation institute |
| rehabilitation program |
| rehabilitation program management |
| rehabilitation programmes |
| rehabilitation programming |
| rehabilitation provider |
| rehabilitation service |
| rehabilitation service center |
| rehabilitation service operations |
| rehabilitation service provider |
| rehabilitation service provision |
| rehabilitation services |
| rehabilitation services firm |
| rehabilitation specialist |
| rehabilitation support |
| rehabilitation therapists |
| rehabilitation therapy |
| rehabilitation therapy firm |
| rehabilitation therapy professional |
| rehabilitation therapy services |
| rehabilitation therapy workflow |
| rehabilitation treatment |
| rehabilitation unit |
| rehabilitation ward |
| rehabilitative care |
| rehabilitative support |
| rehabilitative therapy |
| relapse prevention |
| relapse prevention counselling |
| relapse prevention education |
| relapse prevention research |
| relationship therapy |
| relaxation therapy |
| relief balm |
| relief therapy |
| remote therapy |
| research psychology |
| residential addiction treatment |
| residential addiction treatment service |
| residential behavioral treatment |
| residential rehab |
| residential rehabilitation |
| residential rehabilitation program |
| residential rehabilitation service |
| residential therapeutic care |
| residential therapeutic services |
| residential treatment |
| residential treatment center |
| residential treatment facility |
| residential treatment programs |
| residential-based treatment |
| resiniferatoxin |
| respiratory distress |
| respiratory distress syndrome |
| restrictive pain relief |
| retail medical spa |
| risperidone |
| runners' knee pain relief |
| rx cannabis |
| rylomine |
| schedule iii - v control substances |
| schedule mental-health therapists |
| schizophrenia |
| schizophrenia drugs |
| schizophrenia therapy |
| schizophrenia treatment |
| schizophrenia treatment drugs |
| school based services |
| school-based therapy |
| sciatica pain relief |
| scs therapy |
| sedation tablets |
| sedative |
| sedative medicine |
| selective inhibitors |
| self harm reduction |
| self-hypnosis |
| self-hypnosis program |
| senior care therapy |
| senior citizen mental health |
| sensory therapy |
| serotonin drugs |
| serotonin reuptake inhibitors |
| serotonin supplements |
| severe insomnia control |
| sexual abuse counseling |
| sexual addiction |
| shockwave therapy |
| shockwave treatment |
| short term rehab |
| short term rehabilitation |
| short term rehabilitation care |
| short term rehabilitation stay |
| short term rehabilitative care |
| shoulder pain |
| shoulder pain treatment |
| significant therapy |
| sleep assessment |
| sleep behavior |
| sleep clinic |
| sleep disorder |
| sleep disorder assessment |
| sleep disorder care |
| sleep disorder center |
| sleep disorder cure |
| sleep disorder diagnose |
| sleep disorder diagnostic center |
| sleep disorder diagnostic device |
| sleep disorder diagnostics |
| sleep disorder drugs |
| sleep disorder management platform |
| sleep disorder monitoring |
| sleep disorder patch |
| sleep disorder recovery |
| sleep disorder service |
| sleep disorder testing |
| sleep disorder therapy |
| sleep disorder treatment |
| sleep disorder treatment services |
| sleep drugs |
| sleep issues |
| sleep medication |
| sleep medicine |
| sleep medicine center |
| sleep medicine services |
| sleep monitoring |
| sleep stimulation |
| sleep stimulation technology |
| sleep therapy |
| sleep therapy clinic |
| sleep therapy device |
| sleep therapy equipment |
| sleep therapy machine |
| sleep therapy needs |
| sleep therapy product |
| sleep therapy program |
| sleep therapy resupply |
| sleep therapy services |
| sleep therapy system |
| sleep tracking app |
| sleep treatment |
| sleep treatment device |
| sleep-aid medicine |
| sleeping pills |
| sleeping problems treatment |
| sleeping program |
| sleeping therapy |
| sleepless treatment |
| sleeplessness |
| sleepy therapy program |
| smoking cessation |
| smoking cessation program |
| smoking cessation services |
| smoking cessation treatment |
| smoking habit analysis |
| smoking pills |
| smoking prevention |
| smoking quitting program |
| smoking treatment |
| sober living group |
| sober living home |
| sober living resident |
| sober living space |
| sobriety |
| sobriety management platform |
| sobriety rating |
| social anxiety disorder |
| social behaviors learning |
| social emotional disorder |
| social emotional learning |
| sound therapy |
| sound therapy device |
| sound therapy system |
| special education |
| special education and healthcare |
| special education management system |
| special education services |
| special needs |
| special needs adults |
| special needs assessment |
| special needs children |
| special needs community |
| special needs dentistry |
| special needs education |
| special needs healthcare |
| special needs parenting |
| special needs planning |
| special needs platform |
| special needs program |
| special needs school |
| special needs support |
| special school |
| specialized therapists |
| specialized therapy |
| spectrum disorder |
| spectrum disorder treatment |
| speech language therapy |
| speech therapy |
| spinal pain relief |
| spine relief |
| steroidal-type anti-inflammatory |
| stimulation device |
| stimulation electrode |
| stimulation medical device |
| stimulation therapy |
| stimulator device |
| stress and sleeplessness |
| stress anxiety |
| stress assessment |
| stress capsules |
| stress control |
| stress control mask |
| stress controlling device |
| stress counseling |
| stress disease cure |
| stress disease therapeutics |
| stress disease treatment |
| stress disorder |
| stress disorder treatment |
| stress evaluation |
| stress management |
| stress medication |
| stress recovery |
| stress relief |
| stress relief formula |
| stress relief medicine |
| stress relief service |
| stress relief therapy |
| stress reliever |
| stress relieving drugs |
| stress remediation |
| stress therapy |
| stress treatment |
| structured therapy program |
| student counseling |
| student mental health |
| student suicides |
| suboxone |
| suboxone for opiate addiction |
| suboxone treatment |
| suboxone treatment center |
| substance abuse |
| substance abuse application |
| substance abuse care |
| substance abuse counseling |
| substance abuse counseling services |
| substance abuse disorder treatment |
| substance abuse disorders treatment |
| substance abuse education |
| substance abuse mental health |
| substance abuse prevention education |
| substance abuse problem |
| substance abuse programs |
| substance abuse recovery |
| substance abuse remedy |
| substance abuse service |
| substance abuse testing |
| substance abuse therapy |
| substance abuse treatment |
| substance abuse treatment services |
| substance addiction recovery |
| substance misuse service |
| substance misuse treatment |
| substance use care |
| substance use disorder |
| substance use disorder monitoring |
| substance use disorder outpatient treatment clinic |
| substance use disorder treatment |
| substance use disorder treatment facility |
| substance use services |
| substance use treatment |
| substance-related disorder |
| suicidal behaviors treatment |
| suicidal crisis |
| suicide |
| suicide attempt detection system |
| suicide detection |
| suicide prevention |
| suicide prevention app |
| suicide prevention program |
| suicide prevention service |
| suicide treatment |
| sunlight therapy |
| supplements for anxiety |
| supportive counseling |
| supportive psychotherapy |
| supportive therapy |
| sureprin |
| surgical pain |
| surgical pain treatment |
| swedish massage |
| tailored pain treatment |
| talk therapy |
| talk therapy software |
| targeted therapy |
| technology-assisted behavior |
| teen counseling |
| teen psychology |
| teen treatment center |
| teenage mentalhealth |
| tele behavioral |
| tele therapy |
| tele-therapy |
| telebehavioral health platform |
| telebehavioral health services |
| telecounseling |
| telehealth counseling |
| telemental health counseling |
| telepsychiatry |
| telepsychiatry appointments |
| telepsychiatry care |
| telepsychiatry service provider |
| telepsychiatry services |
| telepsychiatry services provider |
| telepsychology platform |
| telepsychology tools |
| telerehabilitation |
| teletherapy |
| teletherapy platform |
| teletherapy services |
| temperature-induced pain |
| temperature-induced pain reduction |
| temporary pain relief cream |
| tendinopathy |
| tendons pain therapy |
| tendons pain treatment |
| tennis elbow |
| term disability |
| text chat therapy |
| therapeutic cannabis |
| therapeutic care center |
| therapeutic care services |
| therapeutic center |
| therapeutic child care |
| therapeutic clinic |
| therapeutic cooling treatment |
| therapeutic counseling |
| therapeutic counselling |
| therapeutic day treatment |
| therapeutic marijuana |
| therapeutic massage |
| therapeutic massage products |
| therapeutic massage services |
| therapeutic massage system |
| therapeutic school |
| therapeutic service provider |
| therapeutic services company |
| therapeutic services firm |
| therapeutic services provider |
| therapist |
| therapist booking |
| therapist care |
| therapist centric |
| therapist consulting platform |
| therapist finder platform |
| therapist helper |
| therapist matching |
| therapist network |
| therapist recruitment |
| therapist relationship |
| therapist search platform |
| therapist speech |
| therapist staffing agency |
| therapist supervisor |
| therapist training |
| therapist-assisted stretching |
| therapist-care managers |
| therapists |
| therapists consultation |
| therapists network |
| therapists staffing |
| therapy |
| therapy and counseling |
| therapy and counselling |
| therapy and imaging |
| therapy and massage |
| therapy and rehabilitation services |
| therapy app |
| therapy application |
| therapy area |
| therapy assets |
| therapy assistants |
| therapy at home |
| therapy at-home application |
| therapy automation |
| therapy beds |
| therapy boarding school |
| therapy care |
| therapy center |
| therapy center operator |
| therapy centres |
| therapy chamber |
| therapy clinic |
| therapy clinic operator |
| therapy coaching |
| therapy commercialization |
| therapy company |
| therapy compliance |
| therapy concierge |
| therapy consultation |
| therapy consulting |
| therapy conversation |
| therapy coordinator |
| therapy courses |
| therapy decision |
| therapy development |
| therapy development platform |
| therapy device |
| therapy device developer |
| therapy discovery |
| therapy documentation |
| therapy education |
| therapy filters |
| therapy firm |
| therapy for autism |
| therapy for cancer |
| therapy for children |
| therapy for chronic disorder |
| therapy for depression |
| therapy for diabetes |
| therapy games |
| therapy guide |
| therapy instruments |
| therapy lamps |
| therapy management |
| therapy management program |
| therapy management service |
| therapy management services |
| therapy management software |
| therapy management system |
| therapy management tool |
| therapy massage product |
| therapy monitoring |
| therapy music |
| therapy news |
| therapy nursing |
| therapy operations |
| therapy optimization tool |
| therapy partners |
| therapy paths |
| therapy platform |
| therapy practice |
| therapy practicing |
| therapy prediction |
| therapy product |
| therapy product designer |
| therapy product manufacturer |
| therapy products |
| therapy program |
| therapy progress analysis |
| therapy provider |
| therapy provision |
| therapy regimens |
| therapy rehabilitation |
| therapy research |
| therapy rooms |
| therapy school |
| therapy screening |
| therapy selection |
| therapy service provider |
| therapy services |
| therapy services firm |
| therapy services provider |
| therapy session |
| therapy sessions |
| therapy software |
| therapy solutions |
| therapy staffing |
| therapy staffing needs |
| therapy stores |
| therapy studio |
| therapy suites |
| therapy supplies |
| therapy system |
| therapy system assets |
| therapy techniques |
| therapy technologies |
| therapy technology |
| therapy to patients |
| therapy tools |
| therapy toys |
| therapy training service |
| therapy treatment |
| therapy treatment services |
| therapy vectors |
| therapy-assistance |
| therapy-induced dyskinesias |
| therapy-specific programming |
| thermal therapy |
| thermal therapy device |
| thermal therapy technology |
| thought disorder |
| titrating stimulant medication |
| tms |
| tms therapy |
| tobacco alternative |
| tobacco cessation |
| tobacco cessation program |
| tobacco free product |
| tobacco product |
| topical analgesic cream |
| topical anesthetic |
| topical marijuana |
| topical pain medication |
| track behavioral health |
| traditional therapy |
| trained psychologists |
| tramadol based drugs |
| tramadol drugs |
| tramadol generic drug |
| tranquilizers |
| transdermal cannabinoid treatment |
| transdermal stimulation technology |
| transformative therapy |
| trauma counseling |
| trauma distress syndrome |
| trauma therapy |
| traumatic counseling |
| treating back pain |
| treating chronic pain |
| treating headaches |
| treating inflammatory disease |
| treating pain |
| treating psychosis |
| treating sleep disorder |
| treatment and therapy |
| treatment for alcoholic |
| treatment for autism |
| treatment for eating disorder |
| treatment of depression |
| treatment of inflammation |
| treatment of migraine |
| treatment resistant depression |
| treatment resistant depression treatment drugs |
| treatment therapy |
| trigger point therapy |
| triple reuptake inhibitors |
| ultrasound therapy |
| underwater therapy |
| utpatient mental health organization operator |
| uv therapy |
| vagal nerve stimulation |
| vagus nerve stimulation |
| video counseling |
| video games rehabilitation |
| video games therapy |
| video self-therapy app |
| video therapy |
| video therapy sessions |
| virtual counseling therapy |
| virtual counselling |
| virtual reality stress reliever |
| virtual reality therapy |
| virtual rehabilitation platform |
| virtual therapy |
| virtual therapy games |
| virtual therapy program |
| virtual therapy session |
| visual therapy |
| vr clinical therapy |
| vr therapy |
| wave therapy |
| wearable light therapy |
| wearable neuromodulation patch |
| wearable pain relief |
| wearable spinal cord stimulator |
| wearable therapy |
| web therapy |
| whole body cryotherapy |
| wilderness therapy |
| wrist pain |
| wrist pain relief |
| yoga therapy |
